# A senescence-associated signature refines the classification of different modification patterns and characterization of tumor immune microenvironment infiltration in triple-negative breast cancer

**DOI:** 10.3389/fphar.2023.1191910

**Published:** 2023-05-11

**Authors:** Renhong Huang, Han Wang, Jin Hong, Zheng Wang, Jiayi Wu, Ou Huang, Jianrong He, Weiguo Chen, Yafen Li, Xiaosong Chen, Kunwei Shen

**Affiliations:** Department of General Surgery, Comprehensive Breast Health Center, Ruijin Hospital, Shanghai Jiao Tong University School of Medicine, Shanghai, China

**Keywords:** senescence-associated genes, triple-negative breast cancer, modification patterns, tumor immune microenvironment, FAM3B

## Abstract

**Background:** Recent studies have found that senescence-associated genes play a significant role in cancer biological processes. We aimed to analyze the characteristics and role of senescence-associated genes in triple-negative breast cancer (TNBC).

**Methods:** We systematically screened senescence-associated secretory phenotype (SASP) genes based on the gene expression information in the TCGA database. According to the expression levels of senescence-associated genes, TNBC was classified into two subtypes, namely, TNBCSASP1 and TNBCSASP2, using an unsupervised cluster algorithm. We then performed gene expression, enrichment pathway, immune infiltration, mutational profile characterization, drug sensitivity and prognostic value analyses for the two subtypes. The reliability and prognostic predictive utility of this classification model were validated. The most prognostically relevant gene, FAM3B, was comprehensively identified and validated by tissue microarray in TNBC.

**Results:** TNBC was classified into two senescence-associated subtypes, TNBCSASP1 and TNBCSASP2, based on the set of senescence-associated secretory phenotype genes, among which the TNBCSASP1 subtype had a poor prognosis. The TNBCSASP1 subtype was immunosuppressed, with suppressed immune-related signaling pathways and low immune cell infiltration. The effect of the mutation on the TP53 and TGF-β pathways could be related to the poor prognosis of the TNBCSASP1 subtype. Drug sensitivity analysis showed that AMG.706, CCT007093, and CHIR.99021 were potential targeted drugs for the TNBCSASP1 subtype. Finally, FAM3B was a key biomarker affecting the prognosis of patients with triple-negative breast cancer. Compared to normal breast tissue, the expression of FAM3B was reduced in triple-negative breast cancer. Survival analysis showed that overall survival was significantly shorter in triple-negative breast cancer patients with high FAM3B expression.

**Conclusion:** A senescence-associated signature with different modification patterns has critical potential for providing a better understanding of TNBC biological processes, and FAM3B might serve as an applicable target for TNBC therapy.

## Introduction

Triple-negative breast cancer is a special subtype of breast cancer, accounting for approximately 15%–20% of all breast cancers (Garrido-Castro et al., 2019). Compared with other subtypes of breast cancer, triple-negative breast cancer is more aggressive, with an earlier onset age, larger tumor volume, higher histological grade, early recurrence and distant metastasis, and poor overall survival (Garrido-Castro et al., 2019). With advances in research, it has been gradually revealed that triple-negative breast cancer is highly heterogeneous in biological aspects. In recent years, with the rapid development of multiomics technology, the heterogeneity of triple-negative breast cancer has been further confirmed at the genomic level, transcriptome level, and proteome level. Therefore, it is necessary to study different classification methods and identify the specific subtypes of triple-negative breast cancer to provide direction for the development of new targeted therapy strategies.

Cellular senescence is a permanent state of cell cycle arrest accompanied by changes in cell secretory characteristics (Campisi and dʼAdda di Fagagna, 2007). Cellular senescence is a stress response that can be induced by various internal or external damage signals, including telomere dysfunction, oncogene activation, oxidative stress, and persistent DNA damage. Cellular senescence has long been considered a natural antitumor mechanism. Among them, one of the key mechanisms is oncogene-induced senescence (OIS) (Serrano et al., 1997), in which the activation of proto-oncogenes or the inactivation of tumor suppressor genes triggers cell growth arrest. Increasing evidence shows that cell senescence is also closely related to the occurrence and development of tumors. Through the mechanism of the senescence-associated secretory phenotype, senescent cells can act on surrounding tumor cells in a paracrine manner and change the tumor microenvironment to promote the occurrence and development of tumors. In the tumor microenvironment, cellular senescence is immunogenic, enhances MHC-1 antigen delivery, and can activate anti-tumor immune responses mediated by dendritic cells and CD8^+^ T cells (Marin et al., 2023). Based on the above effects, senescent cells have the potential to become emerging markers of cancer (Hanahan, 2022). The senescence-related secretory phenotype is one of the important characteristics of senescence (Rodier and Campisi, 2011). Senescent cells undergo significant changes at the secretion level and secrete many substances dominated by proinflammatory factors, including cytokines, chemokines, growth factors and extracellular matrix proteases. By activating the above transcriptome, senescent cells can enhance their own senescence process in an autocrine manner and send signals to neighboring cells in a paracrine manner, thereby affecting the tissue microenvironment. Senescence cells can enhance macrophage display of senescence characteristics by secreting SASP factors. Senescence macrophages can affect other parts of the immune system to evade immune surveillance and clearance of senescence cells (Prieto and Baker, 2019). In addition, the accumulation of senescent cells in the tumor microenvironment will also promote the release of SASP factors and promote the growth of tumor cells. The relationship between senescence-related secretory phenotypes and tumor development is complex and is manifested by the pleiotropic effects of senescence-related secretory phenotypes. With the development of multiomics technology, the genes that play a key role in cellular senescence have been gradually identified by researchers, and senescence-related genomes have been formed. The proposal of senescence-related genomes provide data support for further research on the relationship between senescence and tumors. Using senescence-related datasets from the literature, a comprehensive pancancer analysis was conducted (Zhao et al., 2022). The results showed that senescence-related genes are widely different in different cancers and that cell senescence has an important impact on the tumor immune microenvironment. Wang et al. (Wang et al., 2022) used the senescence-related gene set to define a cellular senescence score in pancancer and demonstrated that this score can represent the degree of immune activation in the tumor microenvironment and can identify groups with better prognosis. In specific cancers, researchers have also constructed classification models and prognostic prediction models based on senescence gene sets, including clear cell renal cell carcinoma (Lu et al., 2022), hepatocellular carcinoma (Luo et al., 2022), glioblastoma (Tan et al., 2022), etc., and explored the relationship between senescence-related genes and clinical characteristics, tumor microenvironment, immunotherapy, etc. These studies demonstrate the importance of senescence genes in cancer therapy.

The aim of this study was to construct a classification model for triple-negative breast cancer based on the expression profile of senescence-associated secretory phenotype genes and to explore the prognostic value of this classification model. The differences in different senescence-related subtypes were compared at the multiomics level, including gene enrichment pathways, immune infiltration, genomic mutations, drug sensitivity, etc. The classification model was validated on external datasets. Furthermore, based on the senescence-related subtypes of triple-negative breast cancer, the key genes affecting the prognosis of different subtypes were explored, and the prognostic role of these genes in triple-negative breast cancer was analyzed.

## Method and material

### Data collection and processing

The pan cancer expression profiles and survival information, which included 110 triple-negative breast cancer patients from TCGA-BRCA, were retrieved from the XENA datasets (http://xena.ucsc.edu/) (Tomczak et al., 2015). Genomics information, containing copy number variation and SNV of BRCA, was downloaded from The Cancer Genome Atlas (TCGA) (Blum et al., 2018). Three independent TNBC cohorts were used: GSE21653 (252 samples), GSE25066 (205 samples) and GSE103091 (111 samples) (Hatzis et al., 2011; Sabatier et al., 2011; Jézéquel et al., 2015). For cohorts downloaded from public datasets, instructional review board approval or informed consent was not needed. A novel senescence-associated signature was acquired from the Supplementary Material from the Saul et al. search (Supplementary Table S1).

### Unsupervised cluster analysis

First, we performed univariable Cox analysis to filter prognosis-related genes from the senescence-associated signature. Finally, a three-signature-based expression matrix of TCGA-TNBC, including CXCL1, CCL13 and ACVR1B, was maintained to perform unsupervised clustering analysis to identify novel senescence subtypes of TNBC with the use of the ConsensusClusterPlus package (Wilkerson and Hayes, 2010). The optimal cluster number *k* of TNBC was evaluated by the proportion of ambiguous clustering (PAC score) and consensus cumulative distribution function (CDF) curve.

### Differential expression and enrichment analysis

The count mRNA expression matrix of TNBC was used to conduct differential expression analysis through the DEseq2 package and visualized by the EnhancedVolcano package (Love et al., 2014). The threshold was set as abstract log-fold change = 1.5 and *p*-adjusted value <0.01. In addition, common enrichment, including Gene Ontology (GO), gene set enrichment analysis (GSEA) and gene set variation analysis (GSVA), was also performed with the ClusterProfiler package (Yu et al., 2012). For differential expression gene annotation, we downloaded cancer-related hallmarks from the MSigDB dataset and IOBR package (Liberzon et al., 2011).

### Immune infiltration analysis

Five immune-related signature gene sets, containing chemokines, chemokine receptors, MHCs, immunoinhibitors and immune stimulators, were compared between subtypes. Several immune-related deconvolution algorithms, including TIMER, CIBERSORT, QUANTISEQ, MCPCOUNTER, XCELL and EPIC, were adopted to compare the different immune components. The tumor immune dysfunction and exclusion (TIDE) algorithm was further utilized to estimate the immunotherapy response score for TNBC. In addition, the Tracking Tumor Immunotype (TIP) algorithm was also applied to compare antitumor immunity differences between subtypes, which included 7 steps as follows: release of cancer cell antigens (step 1), cancer antigen presentation (step 2), priming and activation (step 3), trafficking of immune cells to tumors (step 4), infiltration of immune cells into tumors (step 5), recognition of cancer cells by T cells (step 6), and killing of cancer cells (step 7) (Xu et al., 2018). The Estimate package was further adopted to verify the immune infiltration degree difference between subtypes.

### Mutation spectrum characteristics

We downloaded genomic mutation, except for germline mutation, profiles of TNBC from TCGA GDC database, then we compared and visualized the difference between subtype through Maftools package (Mayakonda et al., 2018). Multilevel differences in the genomic profile, including onco-pathway, somatic interaction, mutation prognostic impact and drug categories, were also compared by Maftools and ggpubr packages.

### Validation of remodeling results in validation cohorts

After identifying subtype biomarkers of each subtype, we next applied the nearest template prediction (NTP) algorithm to perform remodeling analysis in three independent TNBC cohorts. After estimating each sample’s cluster tendency, we compared the prognostic difference to verify the reproductivity of unsupervised cluster results.

### Potential implications of preclinical treatment agent analysis

For therapeutic sensitivity analysis, we first collected the subtype and mRNA expression of TNBC; then, the cell line’s expression profile and therapeutic information from genomic of drug sensitivity in cancer (GDSC) datasets were also downloaded (Cokelaer et al., 2018). We next applied Ridge’s regression and 10-fold cross validation to identify subtype molecular agents by the pRRophetic package (Geeleher et al., 2014). Half maximal inhibitory concentration (IC50) values were applied to compare different sensitivities between subtypes.

### Clinical data collection and follow-up

The clinicopathological data of the patients in this study were obtained from the Shanghai Jiao Tong University-Breast Cancer Database (SJTU-BCDB). The included clinicopathological features were the following: age, menstrual status, tumor location, tumor size, number of lymph node metastases, TNM stage, histological grade, lymph vascular invasion, Ki-67 index, and adjuvant therapy information. A Ki-67 index >30% was defined as high Ki-67 expression, and a Ki-67 index ≤30% was defined as low Ki-67 expression. All breast cancer patients received regular follow-up in the clinic or by telephone. Patients were followed up every 3 months for 2 years after surgery. Follow-up was performed every 6 months for 3–5 years, and follow-up was performed annually 5 years after surgery until event and death. The follow-up data were recorded and summarized by the breast specialist nurses and clerks in our department. Overall survival (OS) and disease-free survival (DFS) were defined as reported in our previous study (Huang et al., 2022). The last follow-up was in January 2023. Patients’ inclusion and exclusion criteria were described in the Supplementary Figure S2. The detail information of these patients was shown in Supplementary Table S5.

### Immunohistochemistry (IHC) of tissue microarray

IHC staining of FAM3B protein expression in the tissue microarray was performed by incubation with rabbit polyclonal antibodies against human FAM3B antibody (27131-1-AP, Proteintech, 1:200) overnight, followed by incubation with goat monoclonal antibody against rabbit antibody (111-035-003, JACKSON, 1:1,000) for 1 h at room temperature. The immunohistochemical evaluation of FAM3B was employed and analyzed by two individual pathologists, Anqi Li and Miao Ruan, who were blinded to the clinical information of the patients. The immunohistochemical staining results were analyzed by ImageJ processing software. Protein expression was evaluated based on the optical density (OD) value of the images. Immunohistochemical staining was evaluated at the same magnification (×40) in five randomly selected fields of tumor tissue from each patient. After setting a specific threshold for each image, the integrated optical density (IOD) value was calculated in the positive area. The average optical density (AOD) was calculated by the ratio of IOD to the area of the positive area. The average AOD value of the five regions was taken as the result of the AOD value of the patient, which represented the expression level of the markers.

### Statistical analysis

All multiomics dataset processing, plotting and statistical tests were performed using R software (Version 4.1.0). Student’s t tests and Mann–Whitney U tests were applied for continuous variables with normal or skewed distributions. The chi-square test or Fisher’s exact test was utilized to compare categorical variables. Spearman correlation was used to calculate the correlation index between quantitative variables by the corrplot package. Kaplan‒Meier and time ROC curves were depicted by the survival package. All statistical tests were two-sided with a level of significance set as *p* < 0.05.

## Results

### Expression of senescence-associated genes in pancancer

**FIGURE 1:**
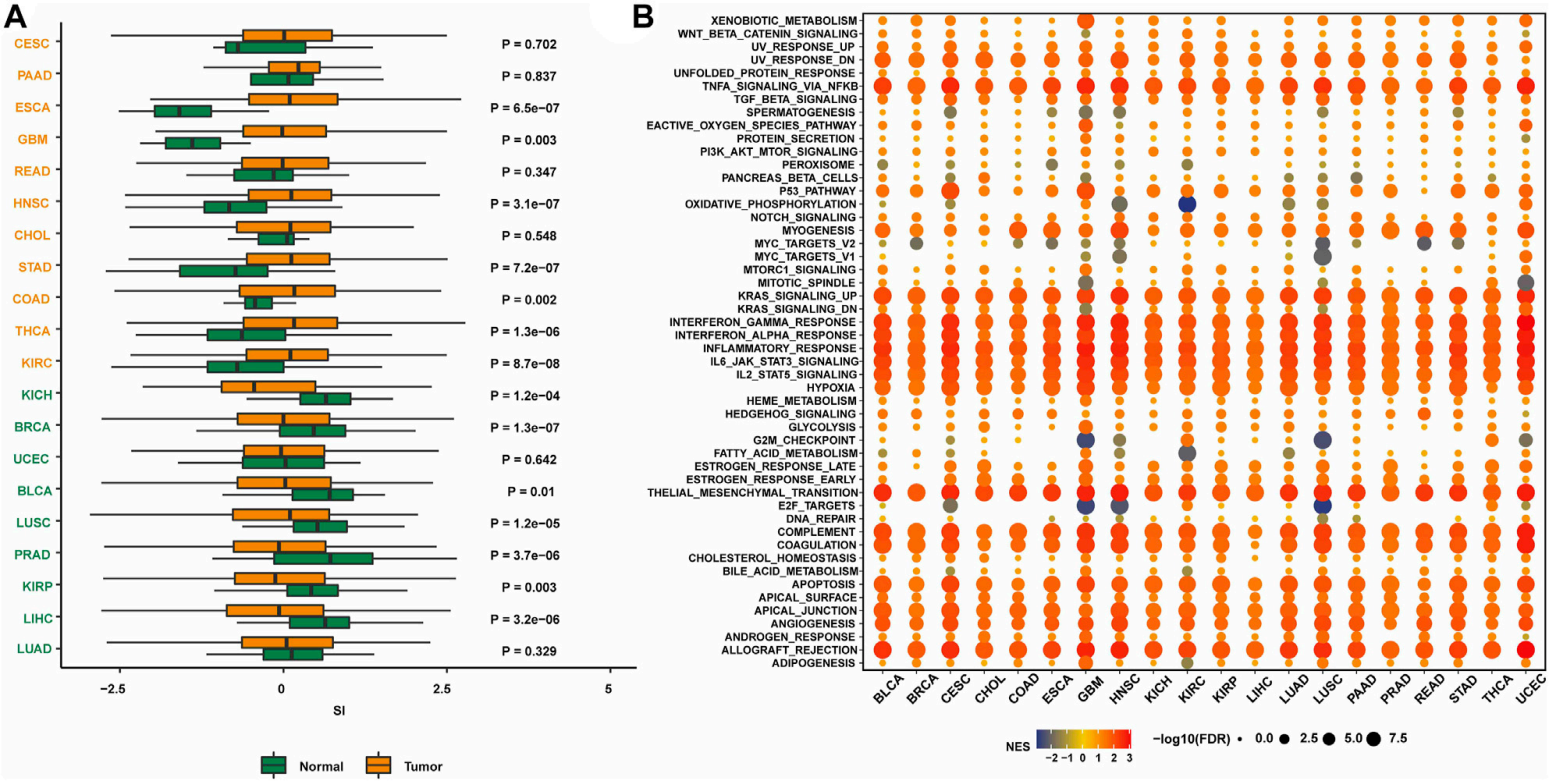
The expression of SASP genes across cancers. **(A)** The landscape of SASP gene set scores (based on NES and *p* values) in 20 cancer types. **(B)** The association between SASP gene set score and expression with HALLMARKS enrichment scores in 20 cancer types.

To initially explore the expression of senescence-related genes in tumors, the expression levels of senescence-related genes in tumor tissues and normal tissues were compared in 20 cancer types (Figure 1A). The results showed that in several cancer types, including breast cancer, the expression of senescence-related genes was lower in tumor tissues than in normal tissues, indicating that the expression of senescence-related genes was suppressed. Activating senescence-related pathways may be a potential way to treat breast cancer.

Next, the enrichment of senescence-related genes in classical tumor pathways was investigated. Senescence-related genes were associated with multiple immune pathways, including the KRAS upregulation pathway, IFNγ response pathway, IFNα response pathway, immune response pathway, IL-6/JAK/STAT3 pathway, and IL-2/STAT5 pathway (Figure 1B). The activation status was consistent in many cancer types. Therefore, targeting senescence-related pathways may activate immune regulation and other pathways, thereby promoting the occurrence and development of tumors.

### Construction and functional enrichment analysis of senescence-associated subtypes in TNBC

**FIGURE 2:**
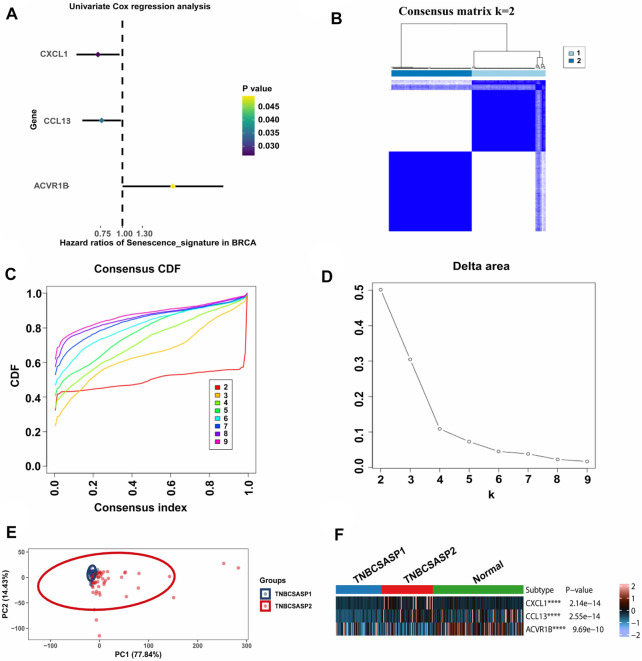
Clustering of SASP genes for the identification of different cancer modification patterns in TNBC. **(A)** Univariate Cox regression analysis of SASP genes in TNBC. **(B)** Consensus matrix heatmap displaying the scale for two cleanly separated clusters. **(C)** CDF plot shows flattening of the consensus index curve for every consensus matrix from two to nine. **(D)** Delta area curve of consensus clustering, which indicates the relative change in area under the CDF curve for each category number k compared to that of k-1. The horizontal axis represents the category number k, and the vertical axis represents the relative change in area under the CDF curve. **(E)** Principal component analysis was carried out based on the results of consensus clustering. **(F)** The expression profiles of SASP regulator genes among the two subtypes and normal tissues.

By analyzing the senescence-related genes, three genes significantly associated with prognosis were screened out among the 125 genes in the senescence-related gene set using univariate Cox regression analysis (*p* < 0.05) (Figure 2A). CXCL1 (*p* = 0.026, HR = 0.723) and CCL13 (*p* = 0.035, HR = 0.761) were protective factors related to prognosis, while ACVR1B (*p* = 0.049, HR = 1.945) was a risk factor related to prognosis. Unsupervised cluster analysis was performed using the Consensus Cluster Plus package in the TNBC population of the TCGA database. According to the cumulative distribution function (CDF) and the proportion of ambiguous clustering (PAC) (Figures 2B–D), by analyzing the distribution of the CDF curve and the change in the area under the curve, the classification reliability was evaluated, and the optimal number of Clusters *k* = 2 was obtained. According to the clustering results, the population in TCGA-TNBC was divided into two senescence-related subtypes, named TNBCSASP1 and TNBCSASP2. The results of principal component analysis (PCA) based on senescence-related genotyping were analyzed (Figure 2E). Further analysis of the gene expression profiles of CXCL1, CCL13, and ACVR1B in the two senescence-related subtypes and normal breast tissues (Figure 2F) showed that CXCL1 and CCL13 were significantly downregulated in the TNBCSASP1 subtype.

**FIGURE 3:**
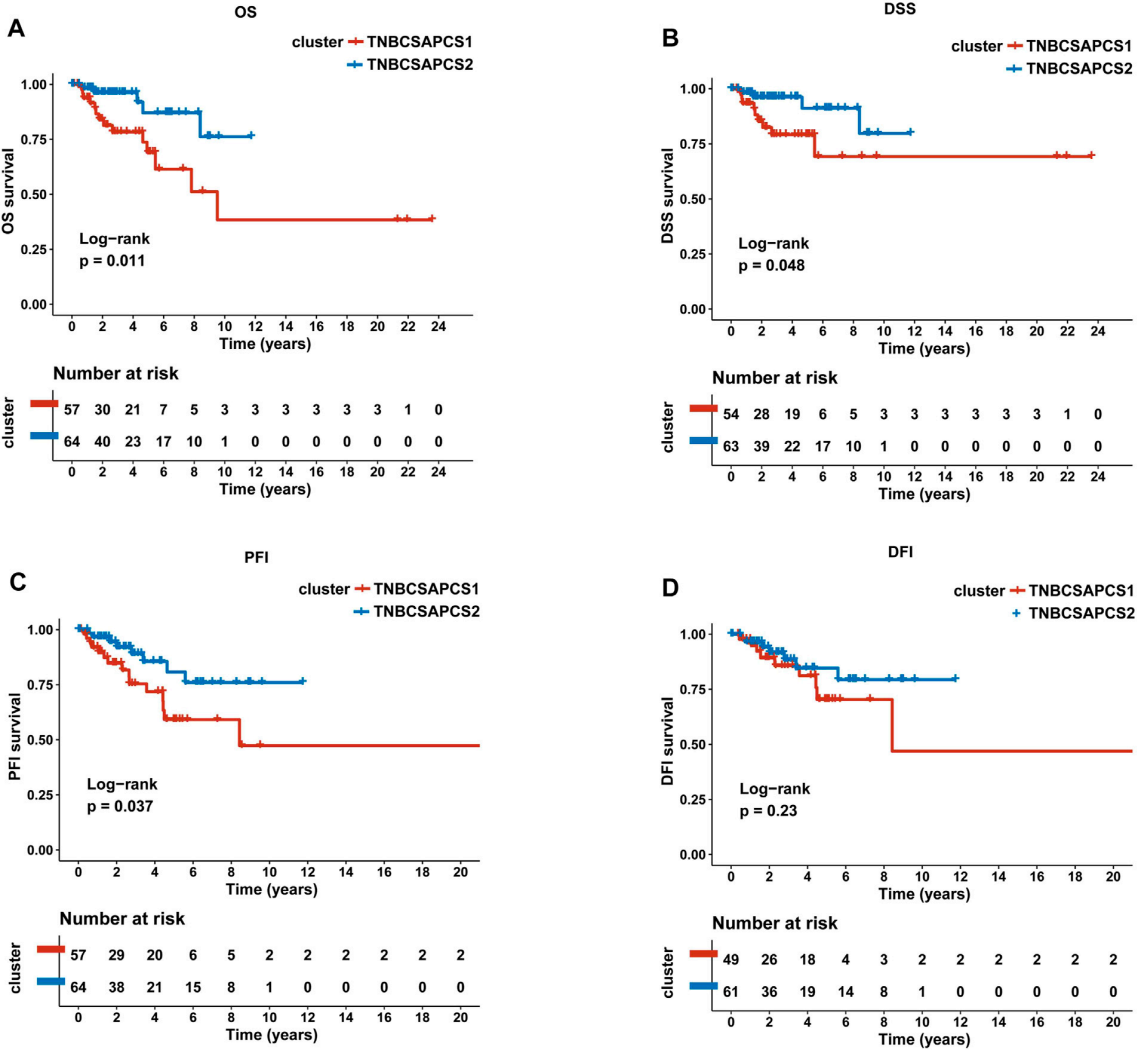
Prognostic value of TNBCSASP1 and TNBCSASP2 in TNBC. Survival analysis for OS **(A)**, DSS **(B)**, PFI **(C)** and DFI **(D)** among the TNBCSASP1 and TNBCSASP2 subtypes of TCGA data.

**FIGURE 4:**
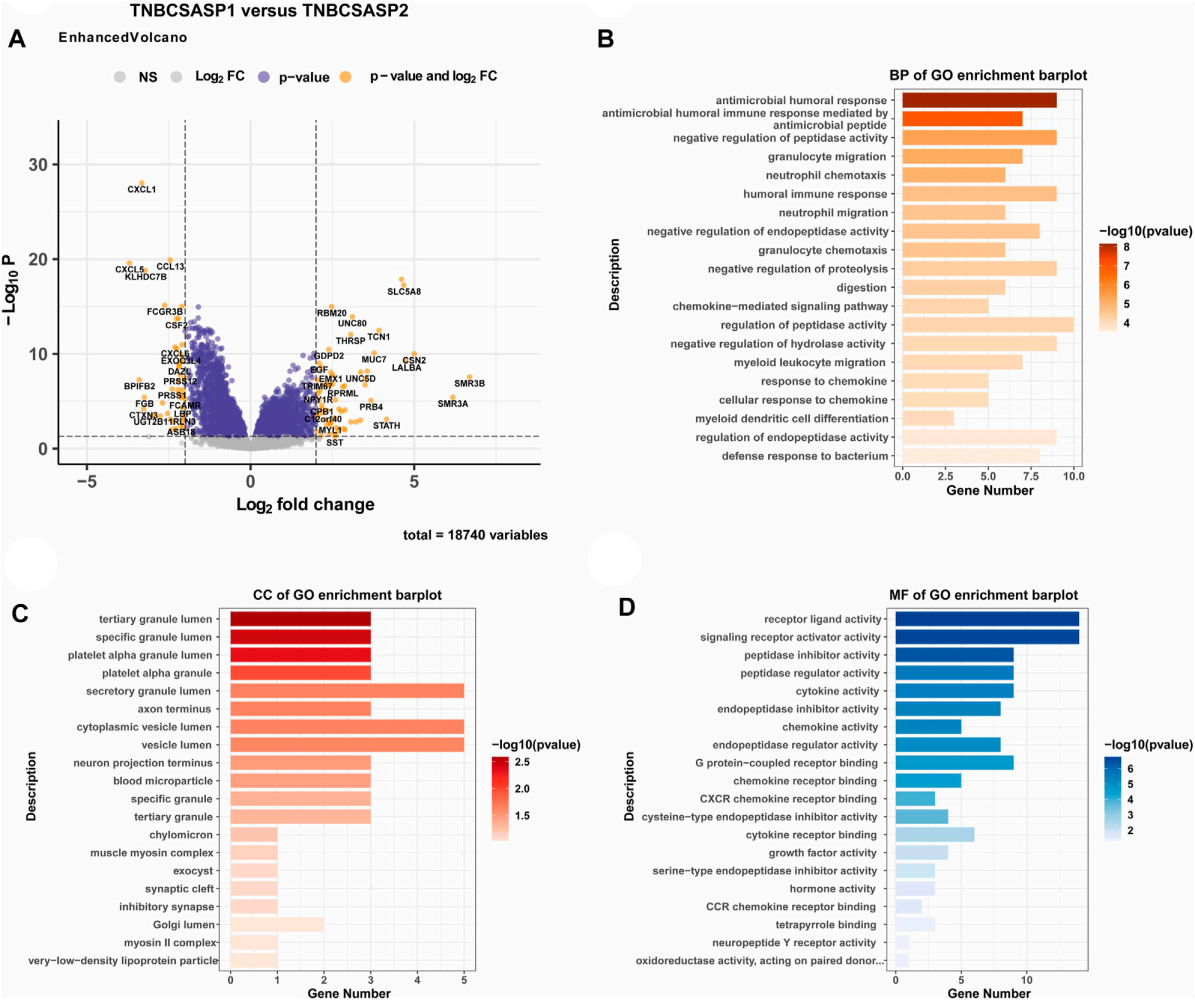
Functional enrichment analysis of the TNBCSASP1 and TNBCSASP2 subtypes. **(A)** Volcano plot of the landscape of gene expression changes between the 2 subtypes. **(B–D)** The GO terms of the BP, CC, and MF categories enriched in the senescence-associated differentially expressed genes. genes.

**FIGURE 5:**
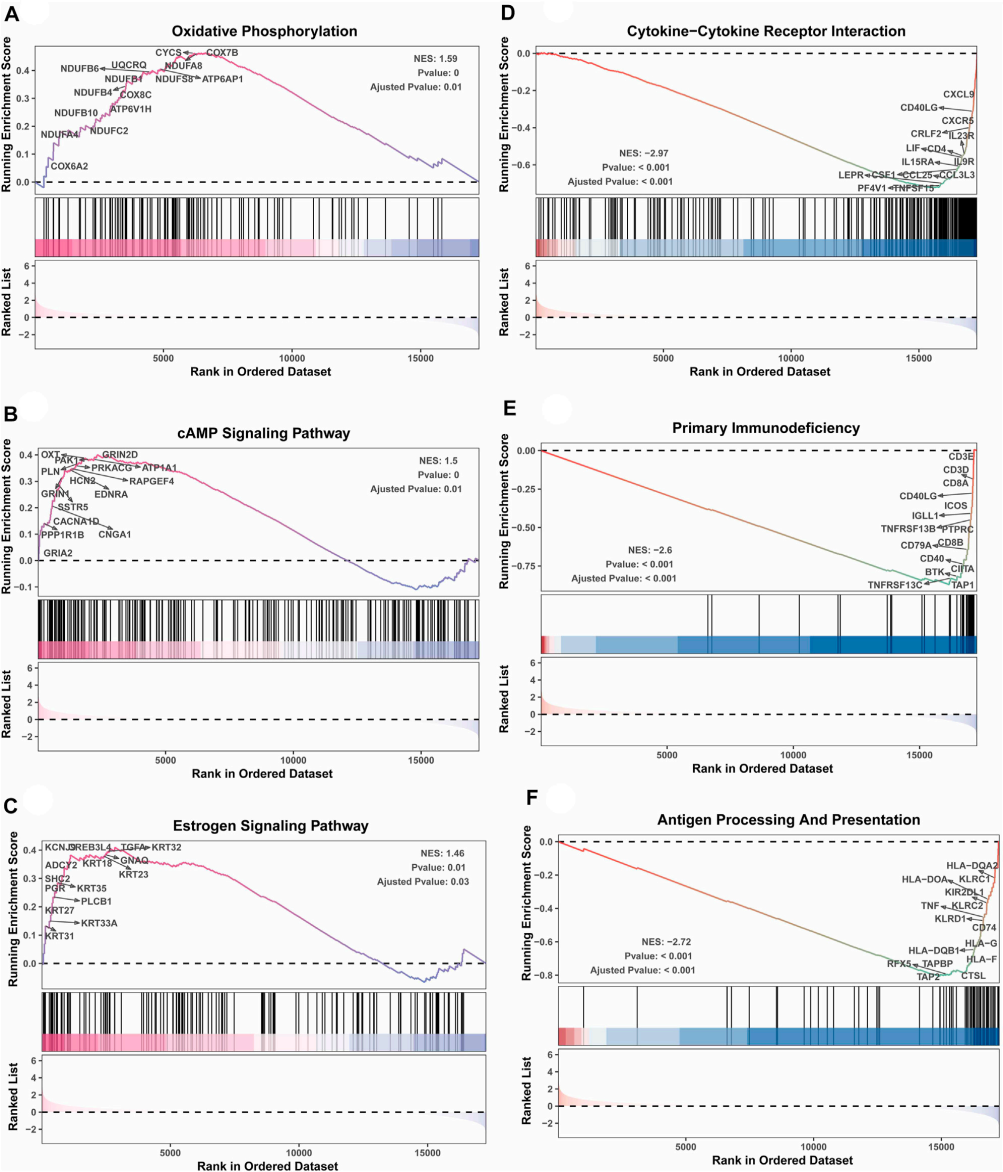
GSEA depicting the enriched pathways of senescence-associated differentially expressed genes in TNBC. These pathways include oxidative phosphorylation **(A)**, cAMP signaling pathway **(B)**, estrogen signaling pathway **(C)**, cytokine‒cytokine receptor interaction **(D)**, primary immunodeficiency **(E)**, and antigen processing presentation **(F)**.

Next, the clinical significance of senescence-related subtyping in terms of prognosis was evaluated by comparing the survival outcomes of the two subgroups. Kaplan‒Meier survival analysis showed that patients in the TNBCSASP1 group had poor survival outcomes (Figure 3), and the OS, DSS, and PFI of the TNBCSASP1 group were significantly lower than those of the TNBCSASP2 group (*p* < 0.05). TNBCSASP classification based on senescence-related genes can predict the prognosis of TNBC patients to a certain extent. Based on the different prognoses of the TNBCSASP1 and TNBCSASP2 groups, it was necessary to further explore the specific biological differences between the two subtypes. The differentially expressed genes of the two subtypes were analyzed using the DEseq2 package (Figure 4). Next, functional enrichment analysis of differentially expressed genes was performed using the ClusterProfiler package. GO (Gene Ontology) enrichment analysis showed that in terms of biological process (BP), differentially expressed genes were mainly involved in antibacterial humoral response, antimicrobial peptide-mediated humoral immune response, negative regulation of peptidase activity and other processes. In terms of cell component (CC), the differentially expressed genes were mainly located in the tertiary granule cavity, specific granule cavity and platelet α granule cavity. In terms of molecular function (MF), the differentially expressed genes were mainly involved in receptor ligand activity, signal receptor activator activity, peptidase inhibitor activity and other functions. GSEA of senescence-related differentially expressed genes showed that oxidative phosphorylation, cAMP signaling pathway, and estrogen signaling pathway were activated, while cytokine receptor interaction pathway, primary immunodeficiency pathway, and antigen processing and presentation pathway were inhibited in TNBCSASP1 subtypes (Figure 5). These results suggest that the TNBCSASP1 subtype tumors may be in a state of poor immune response due to the inhibition of immune-related pathways, leading to a poor prognosis of patients with this subtype.

### Analysis of immune infiltration in senescence-associated subtypes of TNBC

**FIGURE 6:**
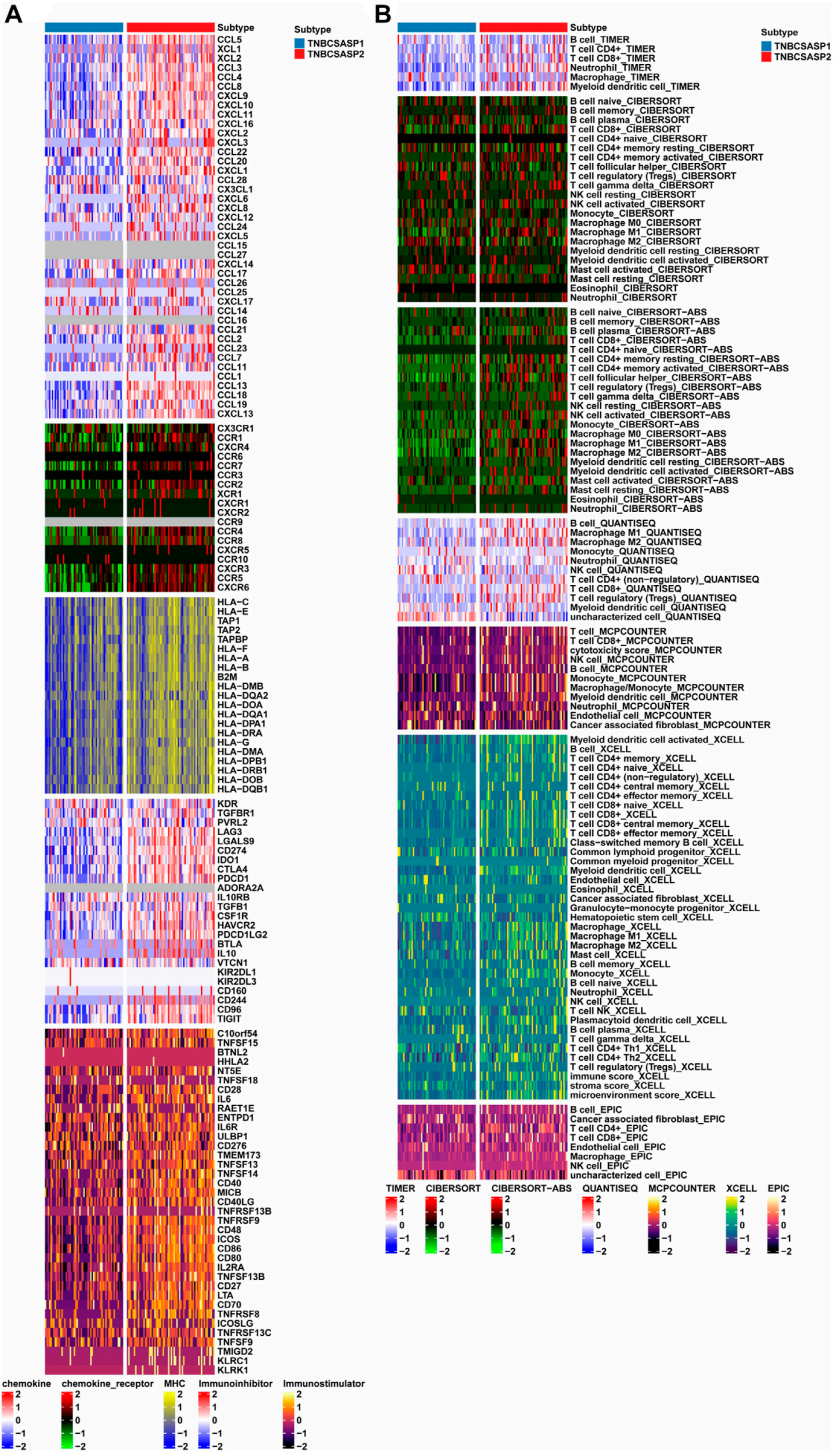
Comprehensive analysis of immune landscapes in TNBCSASP1 and 2 subtypes. **(A)** Heatmap showing differential immune-related genes among the TNBCSASP1 and TNBCSASP2 subtypes. **(B)** Heatmap plot of tumor-related infiltrating immune cells based on TIMER, CIBERSORT, CIBERSORT-ABS, MCPCOUNTER, QUANTISEQ, EPIC, and XCELL algorithms among the TNBCSASP1 and TNBCSASP2 subtypes.

Based on the above functional enrichment analysis results, the TNBCSASP1 and TNBCSASP2 isoforms showed significant differences in immune-related pathways. To further characterize the immune profile of the senescence-related subtypes of TNBC, five immune-related signature gene sets were analyzed (Figure 6A), including chemokines, chemokine receptors, MHC, immunosuppressive factors, and immune stimulating factors. The results showed that CXCL1, CXCL13, CXCL10, CCL13, CCL18, CCL8, IDO1 and other immune-related characteristic genes were significantly downregulated in TNBCSASP1 subtypes. Subsequently, deconvolution algorithms such as TIMER, CIBERSORT, CIBERSORT-ABS, MCPCOUNTER, QUANTISEQ, EPIC and XCELL were used to compare the composition of immune-infiltrating cells in the tumor microenvironment of the TNBCSASP1 and TNBCSASP2 subtypes. The results showed that the TNBCSASP1 subtype had less infiltration of immune cells, including monocytes, macrophages, myeloid dendritic cells, CD8^+^ T cells, and others, than the TNBCSASP2 subtype (Figure 6B). Therefore, based on the results of the above analysis, the TNBCSASP1 isoform can be defined as an senescence-related isoform in an immunosuppressed state.

**FIGURE 7:**
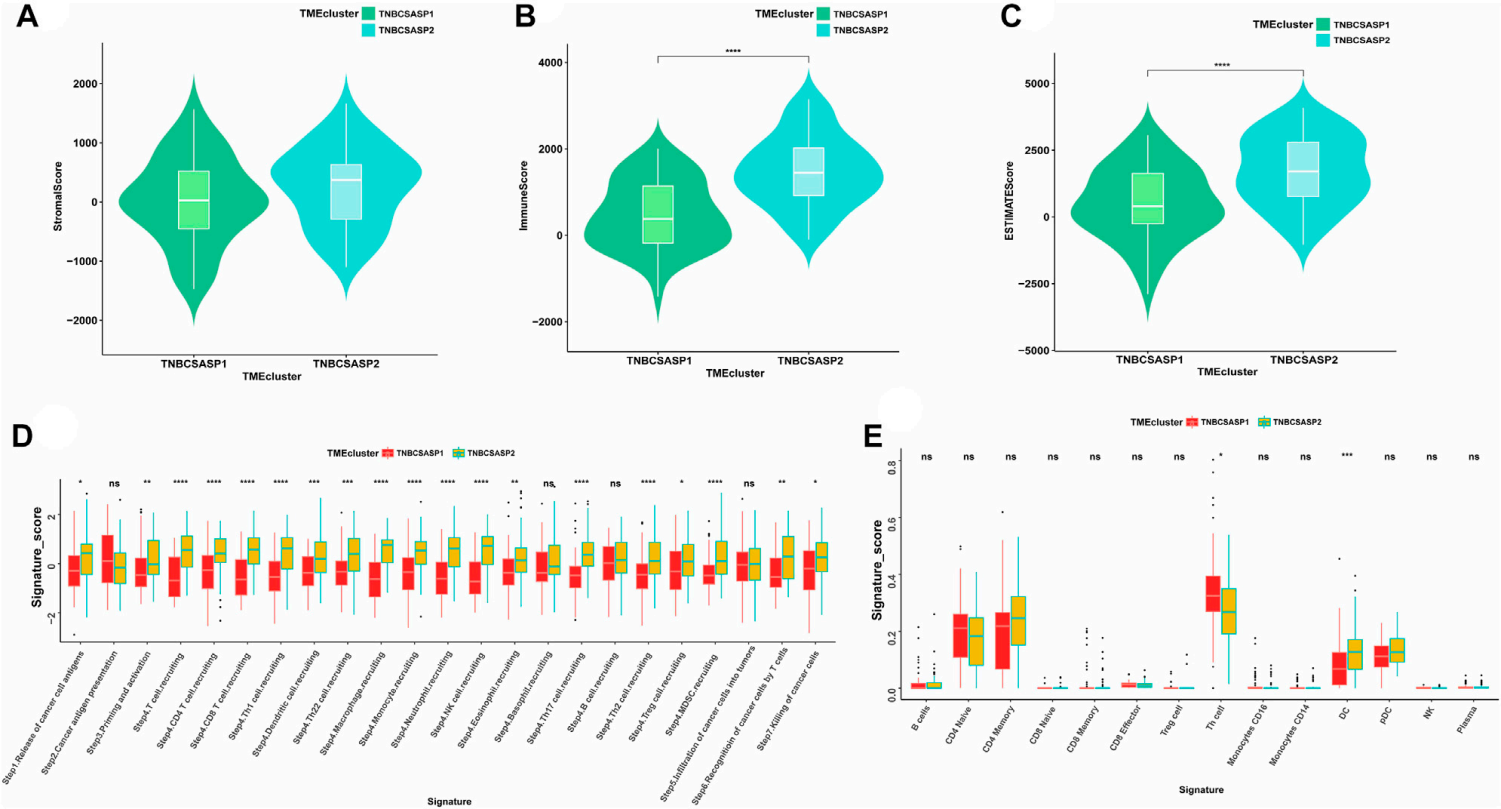
Immune landscapes in TNBCSASP1 and 2 subtypes. Comparison of stromal score **(A)**, immune score **(B)**, and ESTIMATE score **(C)** among the TNBCSASP1 and TNBCSASP2 subtypes. **(D–E)** The immune pathways and anticancer steps among the TNBCSASP1 and TNBCSASP2 subtypes.

Next, the ESTIMATE algorithm was used to explore components of the tumor microenvironment in the two senescence-related subtypes to assess tumor purity (Figures 7A–C). These include the stromal score and immune score, which are used to evaluate the composition of stromal cells and immune cells in tumor samples, respectively. The results showed that the immunoscore and ESTIMATE score of the TNBCSASP1 subtype were significantly lower than those of the TNBCSASP2 subtype, indicating that the TNBCSASP1 subtype had a lower degree of immune cell infiltration and a higher proportion of tumor cells in the tumor microenvironment. Comparing the antitumor activities of the TNBCSASP1 and TNBCSASP2 isoforms (Figure 7D), the TNBCSASP1 isoform was found to be less active during most antitumor steps. Cancer cell antigen release (step 1), CD4 T cells, CDB T cells, macrophages, and other cell recruitment (step 4), T-cell-to-tumor cell recognition (step 6), tumor cell clearance (step 7), etc. Further analysis of the composition of immune-infiltrating cells in the tumor microenvironment of senescence subtypes of TNBC showed that the TNBCSASP1 subtype had a lower infiltration of DCs than the TNBCSASP2 subtype (Figure 7E).

### Somatic mutations in senescence-related subtypes of TNBC

**FIGURE 8:**
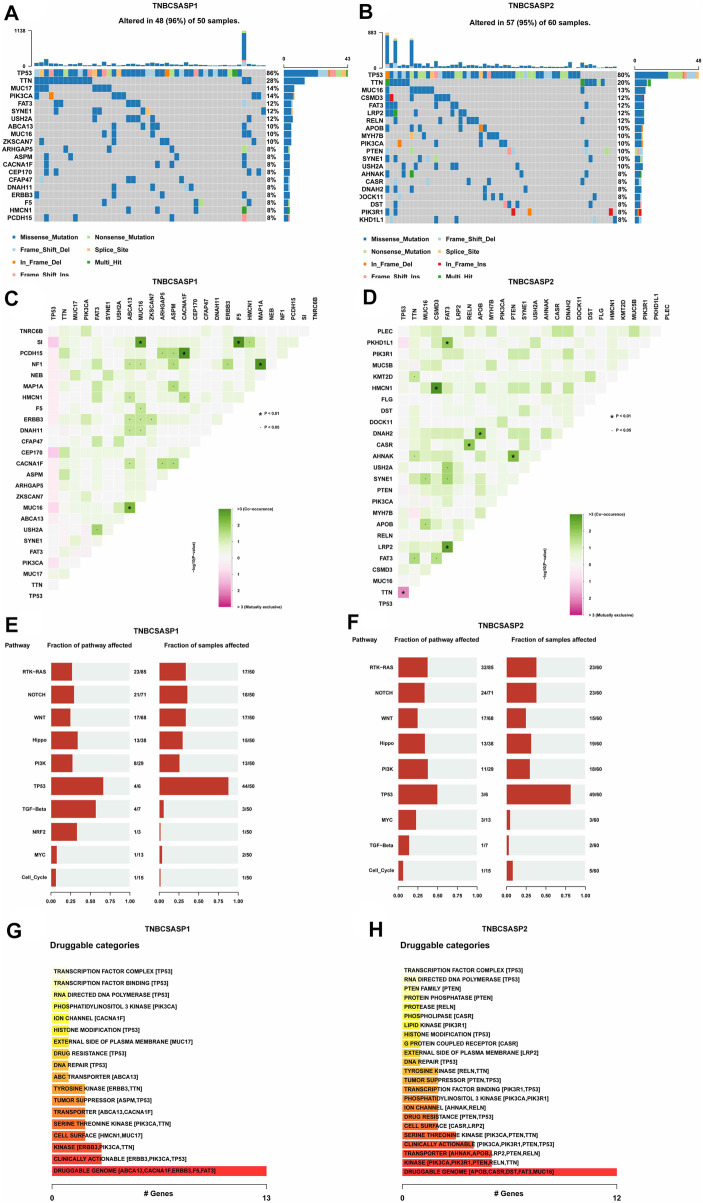
Profiles of somatic mutations and potential targets among the TNBCSASP subtypes. **(A,B)** Waterfall plot showing the mutation patterns of the top 20 most frequently mutated genes. **(C,D)** Cooccurring mutations in TNBCSASP1 and TNBCSASP2. **(E,F)** The fraction of pathways or samples of oncogenic signaling pathways among TNBCSASP1 and TNBCSASP2. **(G,H)** Potential druggable gene categories from the mutation dataset among TNBCSASP1 and TNBCSASP2.

Based on the genome mutation spectrum of TNBC in the TCGA GDC database, Maftools and ggpubr packages were used to analyze the differences in somatic mutations between the TNBCSASP1 and TNBCSASP2 subtypes and to explore the potential carcinogenic factors in the two senescence subtypes. We also identified the top 20 most frequently mutated genes in the two senescence subtypes, with similar mutation rates in the two groups (96% vs. 95%) and high-frequency mutations in TP53 and TTN. MUC17, PIK3CA, ABCA13, ZKSCAN7 and other genes had higher mutation frequencies in the TNBCSASP1 subtype than in the TNBCSASP2 subtype (Figures 8A, B). The somatic comutations of the two senescence subtypes were shown (Figures 8C, D). The TNBCSASP1 subtype had comutations in MUC16-SI, ABCA1-MUC16, CACNA1F-PCDH15, F5-SI, and MAP1A-NF1 (*p* < 0.01). There were comutations of CSMD3-HMCN1, FAT3-PKHD1L1, FAT3-LRP2, RELN-CASR, APOB-DNAH2, and PTEN-AHNAK (*p* < 0.01) in the TNBCSASP2 subtype. TP53-TTN mutations were mutually exclusive. Subsequently, somatic mutations in common tumor-related pathways in both subtypes were evaluated, including the RTK-RAS, NOTCH, WNT, Hippo, PI3K, TP53, TGF-Beta, NRF2, MYC, and Cell Cycle pathways. The results showed that TP53 and TGF-beta pathway receptor cell mutations had the greatest impact on the TNBCSASP1 subtype, whereas TP53, PI3K, and RTK-RAS pathway receptor cell mutations had the greatest impact on the TNBCSASP2 subtype (Figures 8E, F). Next, based on data from the somatic mutant gene set, the DGIdb database and the Maftools package were used to analyze drug-gene interactions in the two senescence-related subtypes to identify potential drug-target genes (Figures 8G, H). The drug target genes of the two senescence-related subtypes could be divided into 18 and 23 types, including druggable genomes, clinically operable genomes, kinase genomes, transport-related genomes, etc.

### Drug sensitivity analysis of senescence-related subtypes in TNBC

**FIGURE 9:**
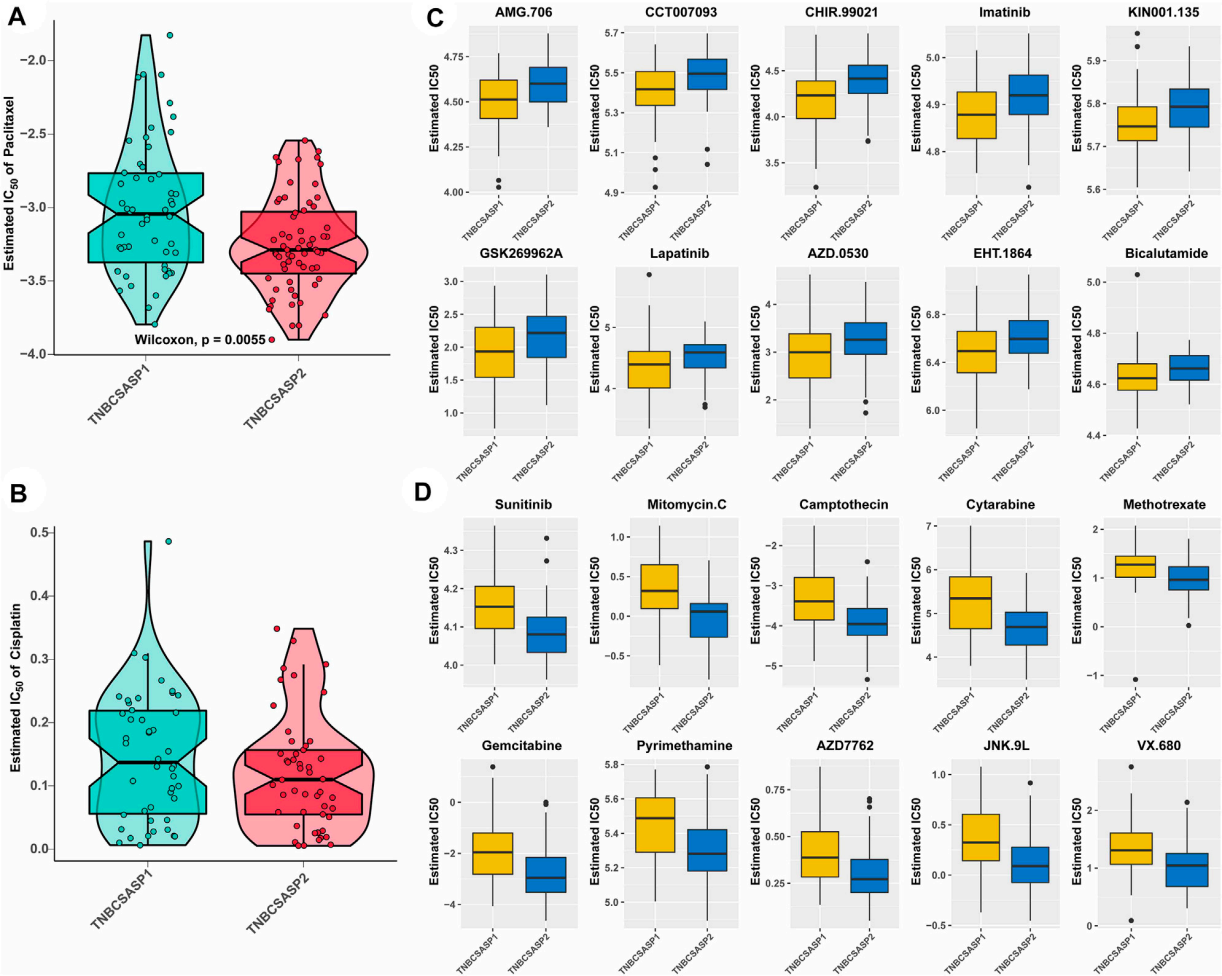
Drug sensitivity analysis of the TNBCSASP subtypes. **(A,B)** Estimated IC50 of the indicated molecular-targeted drugs among TNBCSASP1 and TNBCSASP2. **(C,D)** Estimated IC50 of the potential molecular inhibitors in TNBCSASP1 and TNBCSASP2.

The analysis of drug sensitivity to chemotherapy agents (Figures 9A, B) showed that the semi-inhibitory concentration of the TNBCSASP1 subtype was higher when treated with either paclitaxel or cisplatin, indicating that the TNBCSASP1 subtype was less sensitive to chemotherapy agents, which is consistent with the previous results of worse prognosis of the TNBCSASP1 subtype. We further analyzed the drug responsiveness of the TNBCSASP1 and TNBCSASP2 isoforms to molecular inhibitors and presented the top 10 potential drugs with the most significant therapeutic differences between the two senescence-related isoforms (Figures 9C, D). TNBCSASP1 was more sensitive to AMG.706, CCT007093, and CHR.99021, while TNBCSASP2 was more sensitive to sunitinib, mitomycin C, and camptothecin. It can be predicted that drugs such as AMG.706, CCT007093, and CHIR.99021 are expected to become new development targets for TNBCSASP1, a subtype with poor prognosis, and provide more precise treatment strategies for TNBC patients.

### The prognostic value of senescence-related subtypes and the hub gene FAM3B in TNBC

**FIGURE 10:**
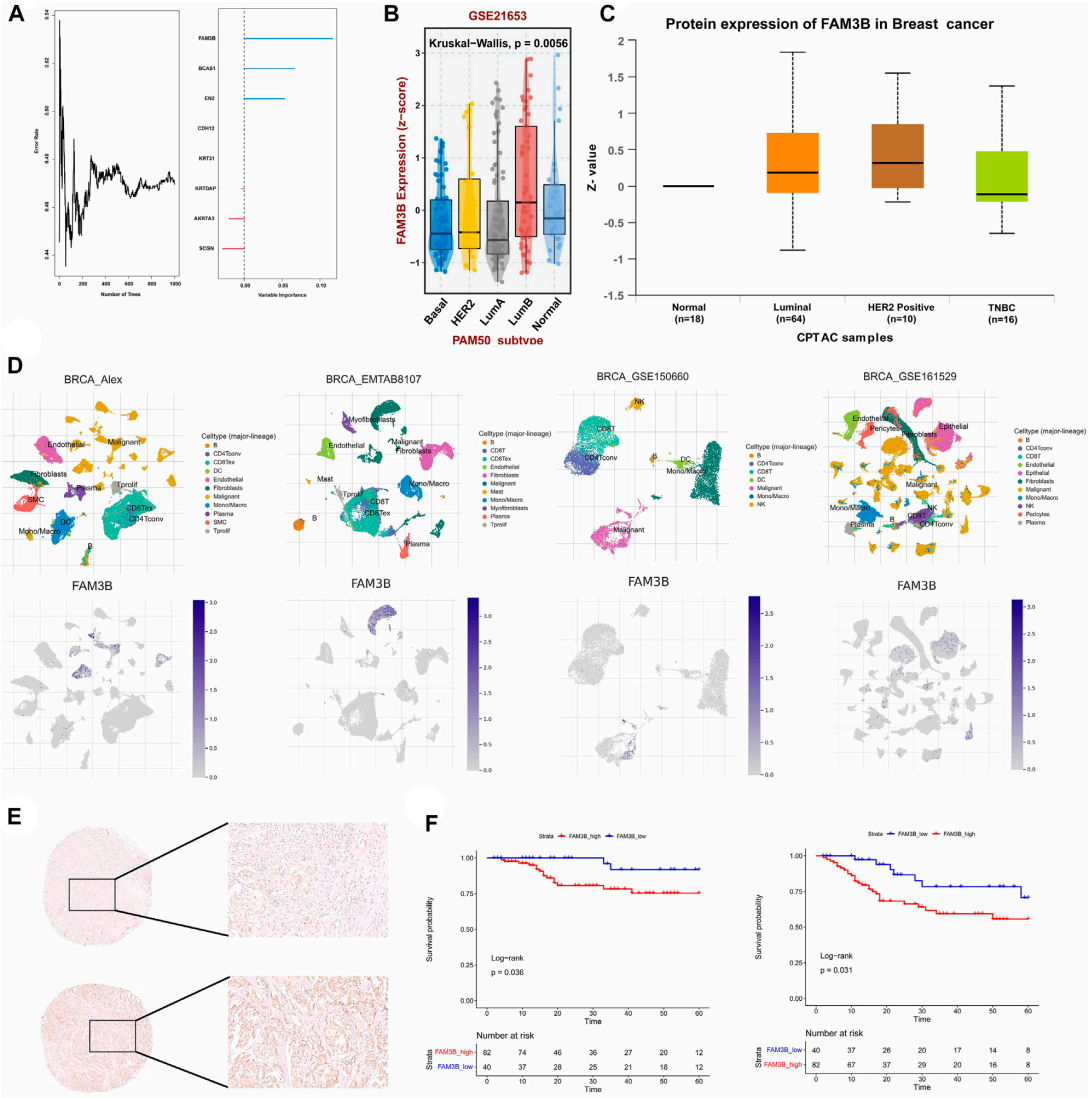
The hub gene FAM3b and its prognostic value in TNBC. **(A)** Number of trees showing the importance proportion of SASP regulator genes. **(B)** FAM3B expression in paired tumor and normal tissues in BRCA from the TCGA database. **(C)** FAM3B expression according to the molecular subtypes of breast cancer. **(D)** UMAP plot of intratumoral immune cells and FAM3B showing the correlation between infiltration of different immune cells and FAM3B expression in the Alex, EMTAB8107, GSE150660 and GSE161529 datasets. **(E,F)** The impact of FAM3B on OS and DFS in breast cancer tissue microarray using Kaplan-Meier analysis.

To confirm the reliability of the senescence-related classification, external datasets were used to validate the classification model. The independent TNBC cohort GSE103091 was divided into two distinct subtypes using the nearest template prediction (NTP) algorithm using the senescence subtype-specific genes identified in the TCGA-TNBC dataset and the classification model reconstruction (Supplementary Figure S1A). A subsequent survival analysis showed that the TNBCSASP1 subtype had a significantly lower survival rate than the TNBCSASP2 subtype (Supplementary Figure S1B, *p* = 0.007), which was consistent with the results of the previous analysis. Therefore, the reliability and stability of senescence-related typing were verified. Based on the above two subtypes, we identified the key differentially expressed gene FAM3B. We used random forest algorithm to identify the gene FAM3B which is most relevant to the prognosis of TNBC (Figure 10A). The expression of FAM3B in triple-negative breast cancer was further analyzed. The relevant data in the GSE21653 cohort were obtained, and the expression level of FAM3B in different subtypes was analyzed based on PAM50 typing (Figure 10B). Compared with normal tissues, the expression level of FAM3B in basal-like subtypes was lower. In addition, proteomic information was obtained from the CPTAC database, and the results similarly showed that the expression level of FAM3B was lower in triple-negative breast cancer tissues (Figure 10C). Single-cell analysis of triple-negative breast cancer was performed in different independent datasets, and FAM3B expression in different cells was investigated (Figure 10D). The results of the analysis of different datasets all showed that FAM3B was specifically highly expressed in malignant epithelial cells in the triple-negative breast cancer population.

From January 2009 to March 2021, 1469 patients with triple-negative invasive breast cancer underwent surgery at our center, and 122 patients were finally included for survival analysis in the study. Univariate analysis of clinicopathological features of patients with different FAM3B expression levels was further performed, and the results showed that there were no significant differences between the FAM3B high expression group and the FAM3B low expression group in each clinicopathological feature (Supplementary Table S2). Univariate analysis of clinicopathological features for predicting prognosis showed that lower clinicopathological stage was associated with longer OS. Higher clinicopathological stage and lymph node metastasis were associated with DFS events. Low FAM3B expression was associated with longer OS and DFS. Other pathological factors did not predict the prognosis of patients (Supplementary Tables S3, S4). Further Kaplan‒Meier survival analysis of the FAM3B high and low expression groups showed that OS (*p* = 0.036) and DFS (*p* = 0.031) in the FAM3B high expression group were significantly lower than those in the FAM3B low expression group (Figures 10E, F). Survival analysis showed that high FAM3B expression was associated with poor prognosis in triple-negative breast cancer.

## Discussion

Triple-negative breast cancer (TNBC) is a subtype of breast cancer with poor prognosis due to its high invasiveness and lack of specific therapeutic targets. As triple-negative breast cancer is highly heterogeneous, it is necessary to characterize different molecular subtypes from a variety of biological perspectives to identify patient risk stratification, predict disease prognosis, and provide potential therapeutic targets.

Based on the existing senescence-associated secretory phenotype gene sets, we constructed a senescence-related classification model for TNBC and defined the two groups as the TNBCSASP1 and TNBCSASP2 subtypes. Survival analysis and multiomics analysis showed that the TNBCSASP1 subtype tumors were in an immunosuppressed state. Immune-related pathways were inhibited, the expression of immune-related factors was lower, the number of immune infiltrating cells was lower, and the TNBCSASP1 subtype showed lower antitumor activity and worse prognosis. Based on the above two subtypes, we identified the key differentially expressed gene FAM3B. Compared with normal breast tissue, FAM3B expression was different in different subtypes of breast cancer. The results showed that high expression of FAM3B in TNBC tumor tissues was related to poor prognosis in patients, suggesting that FAM3B may play a carcinogenic role in TNBC.

Cellular senescence is an important mechanism to maintain tissue homeostasis and aims to eliminate the stress response of damaged cells. Cellular senescence can be induced by damage factors such as DNA damage, reactive oxygen species, activation of oncogenes and inactivation of tumor suppressor genes (Herranz and Gil, 2018). However, recent studies have found that cellular senescence plays an important role in the tumor-promoting process and has been included as one of the emerging markers of cancer (Hanahan, 2022). SASP is characterized by the secretion of many inflammatory factors involved in the immune response, and its dynamic components affect the tumor microenvironment and participate in the regulation of the immune response. SASP can continuously secrete a variety of inflammatory factors, maintain a low adaptive immune response in the tissue microenvironment, and form a “chronic inflammatory” environment (López-Otín et al., 2013). Important related factors include IL-1, IL-6, GM-CSF, IFNγ, TNF and CRP (Zinger et al., 2017). *In vitro* experiments showed that the senescence-related proinflammatory cytokines IL-6 and IL-8 were important for maintaining the invasive properties of the triple-negative breast cancer cell line MDA-MB-231. In a study of the breast cancer population (Knüpfer and Preiss, 2007), IL-6, as a key inflammatory factor, plays an important role in promoting tumor progression, and high circulating levels of IL-6 and age are associated with poor prognosis in breast cancer patients. *In vivo* experiments on breast cancer (Wang et al., 2019), the number of MDSs in the tumor microenvironment of senescence mice was significantly increased, and depletion of MDSCs significantly reduced tumor growth in senescence mice. *In vitro* experiments confirmed that these tumor-specific MDSCs had a highly immunosuppressive effect. In addition, factors in the tumor microenvironment can also induce T-cell senescence and inhibit the immune response. Preclinical studies have shown that the senescence of CD8^+^ T cells plays a key role in the development of breast cancer (Onyema et al., 2015). According to our study, the degree of infiltration of CD8^+^ T cells with immune response function in the TNBCSASP1 subtype was significantly reduced, reflecting the immunosuppressive state of this subtype. On the other hand, the expression of proinflammatory factors and immunosuppressive MDSCs was also generally low in this subtype. TNBCSASP1 subtype has an immune desert phenotype and is more likely to be defined as a “cold tumor” in which effector T cells are unable to infiltrate into the tumor microenvironment, making it difficult for them to exert anti-tumor immune effects. Activating immune cells infiltration may be an effective therapeutic strategy to address the immune escape mechanism in TNBCSASP1 subtype. Since senescent cell types, senescence-inducing factors, tumor progression and other factors affect the pleiotropic effects of SASP, the specific mechanism of SASP on the TNBCSASP1 subtype tumor microenvironment needs to be further explored. Based on the results in this study, we identified TNBCSASP1, a subtype with a worse prognosis based on the difference in expression of SASP-related genes, whose expression of immune-related factors and immune cell infiltration were both at a lower level, indicating that this subtype is in an immunosuppressive state. It can be hypothesized that the difference in expression of SASP factors to some extent makes the two subtypes have different immune microenvironment, which involves the regulation of inflammatory factors, crosstalk of related pathways and gene mutations. The specific mechanism needs to be further investigated.

In addition to the stage of tumor development, senescence also affects the therapeutic response of diseases through its complex mechanism in the treatment stage. Antitumor therapies, including chemotherapy and radiotherapy, can induce cellular senescence in tumor tissues and normal tissues and cause the accumulation of senescent cells (Roberson et al., 2005), namely, therapy-induced senescence (TIS). Senescence-related markers such as p16INK4a, p21, p53 and SA-β-gal can be detected in the tumor tissues of breast cancer patients receiving chemotherapy (te Poele et al., 2002). Another study (Sanoff et al., 2014) showed that cytotoxic chemotherapy induced cellular senescence in hematopoietic tissues of breast cancer patients and prolonged the increase in SASP factors VEGFA and CCL2 levels. In addition, targeted therapies can induce tumor cell senescence. CDK4/6 inhibitors (Goel et al., 2016) can reverse the resistance of HER2-positive breast cancer to anti-HER2-targeted therapy and induce tumor cells to enter cell cycle arrest and have a senescent cell phenotype by inhibiting Rb and S6RP activity. The above evidence suggests that TIS is one of the mechanisms by which antineoplastic therapy works and that cellular senescence is an outcome produced by antineoplastic therapy. However, related studies have shown that TIS cells can produce SASP with cancer-promoting and immunosuppressive functions, which affects the clinical outcome of patients. In non-small cell lung cancer (Wang et al., 2013), TIS is associated with lower overall survival. In the p53 wild-type breast cancer model (Jackson et al., 2012), chemotherapy-induced cell senescence causes tumor regression, and the accumulated senescent cells can secrete cancer-promoting SASP, leading to early recurrence of breast cancer. After neoadjuvant chemotherapy (Morales-Valencia et al., 2022), the exposure of residual breast cancer cells to SASP can lead to the upregulation of intracellular LCN2, enhance tumor invasiveness and is related to tumor chemotherapy resistance. In another study (Muñoz et al., 2019), therapy-induced senescent breast cancer cells could evade immune clearance by paracrine inhibition of NKG2D receptor-mediated immune surveillance. In conclusion, TIS has both antitumor and protumor effects in tumor treatment. Therefore, more studies are needed to quantify the degree of cellular senescence and further evaluate the long-term role of cellular senescence in antitumor therapy.

Current studies have shown that senescence plays an important and complex role in tumors. Therefore, it is necessary to develop new therapeutic regimens for senescence-related targets and incorporate related therapies into future antitumor treatment strategies. In our study, triple-negative breast cancer patients with different senescence subtypes had different drug sensitivities, and small molecule drugs such as the VEGFR inhibitor AMG.706, PPM1D inhibitor CCT007093, and GSK-3α/β inhibitor CHR.99021 were more effective in the TNBCSASP1 subtype. It is expected that senescence-related genes will be used as targets for the development of new drugs in the future. At present, therapies targeting senescent cells have been gradually studied, including the elimination of senescent cells and regulation of senescent cell phenotype. Senolytic drugs are a class of drugs that eliminate senescent cells. Drugs such as ABT-737 and ABT-263 can induce senescent cells to initiate an apoptotic program by inhibiting the activity of BCL-2 family members. *In vivo* experiments have shown (Leverson et al., 2015) that the addition of the BCL-2 family inhibitor A-1331852 enhances the therapeutic effect of docetaxel in a triple-negative metastatic breast cancer model. Senomorphism plays a role by regulating the characteristics of senescent cells, including regulating the SASP and changing the state of cellular senescence. Targeting the mTOR pathway using rapamycin (Zhang et al., 2018) reduces the secretion of the protumorigenic SASP and prevents cellular senescence. Inhibitors of the p38MAPK/MK2 pathway can reduce SASP secretion, thereby inhibiting tumor metastasis in the TIS breast cancer mouse model (Murali et al., 2018). These two approaches to targeting senescence inhibit the tumor-promoting effects of senescence based on different mechanisms. In addition, some new senescence-related markers have also been identified in recent studies, which will help to develop new senescence-related therapeutic targets. Differentiated Embryonic Chondrocyte Gene 1 (DEC1) is one of the target genes of p53, which mediates cell senescence by regulating the phosphorylation of Rb2 protein and acting on the Rb pathway. *In vitro* experiments showed that overexpression of DEC1 could arrest the cell cycle and induce cell senescence. DEC1 downregulation can attenuate DNA damage-induced cellular senescence (Qian et al., 2008). During the progression from normal breast to breast cancer (Chakrabarti et al., 2004), increased expression of DEC1 was observed, suggesting that DEC1 may contribute to the progression of breast cancer to an aggressive phenotype. Human tumor necrosis factor-related apoptosis-inducing ligand receptor 4 (TRAFE-R4) is also one of the target genes of p53. p53 acts on TRAFE-R4 to inhibit its induction of apoptosis. It has been found (Ganten et al., 2009) that breast cancer patients with high expression of TRAFE-R4 have reduced overall survival and disease-free survival. Therapeutic regimens targeting senescence have potential application value, and the development of related drugs still needs to be further verified by experiments.

FAM3B plays an important role in tumor development, disease prognosis, and drug resistance. He et al. (2019) demonstrated that the expression of FAM3B in human esophageal squamous cell carcinoma (ESCC) was higher than that in adjacent tissues, its expression level was significantly related to the clinical stage of ESCC patients, and the high expression level of FAM3B was related to the poor prognosis of patients. Further studies confirmed that FAM3B overexpression could inhibit ESCC cell death by regulating the AKT-MDM2-p53 signaling pathway and affect the epithelial-mesenchymal transition process by regulating Snail and E-cadherin to promote ESCC cell migration and invasion. Cisplatin is one of the main chemotherapy drugs for patients with gastric cancer, but it easily leads to drug resistance during treatment. Song and Duan (2020) found that FAM3B expression was upregulated in cisplatin-resistant gastric cancer cell lines, and FAM3B overexpression affected the epithelial-mesenchymal transition process and induced drug resistance in gastric cancer cells by regulating Snail. Knockdown of FAM3B increased the drug sensitivity of drug-resistant cell lines. Alternatively, the potential role of FAM3B in tumor suppression has been reported. Liao et al. (Liao et al., 2022) used public databases to explore the feasibility of the FAM3 family as a prognostic factor for head and neck squamous cell carcinoma, and the analysis results showed that the low expression level of FAM3B in head and neck squamous cell carcinoma was related to poor prognosis of patients. FAM3B may be related to the increase in immune cell infiltration in the tumor microenvironment, the inhibition of the epithelial-mesenchymal transition process and cytochrome P450 and other targets. In a study on bladder cancer, FAM3B was listed as one of the immune-related genes involved in the construction of a prognosis-related index for bladder cancer, indirectly indicating the association of FAM3B with tumor immunity (Tian et al., 2020).

This study still has some limitations. First, the sample size in this report is limited, and the findings are not fully representative of the overall population of triple-negative breast cancer. Second, the results of this study are based on multiomics bioinformatics analysis, and further experimental verification is needed to explore the specific mechanism of senescence. In addition, the classification model obtained in this study may be affected by some confounding factors, such as race and region, so more independent datasets are needed to validate the classification model. This retrospective study of FAM3B is based on the clinical data of a single center. Complete follow-up data is limited, and there is a bias in population selection. In addition, this study only preliminarily described the expression of FAM3B in triple-negative breast cancer and its effect on prognosis, and the specific mechanism needs to be explored by basic experiments to evaluate the reliability of the results of this study.

Therefore, based on the expression profile of senescence-related secretory phenotype genes, this study created a classification model for TNBC and explored the differential biomarkers of senescence subtypes, which can provide theoretical guidance for the treatment of TNBC.

## Data Availability

The datasets presented in this study can be found in online repositories. The names of the repository/repositories and accession number(s) can be found in the article/Supplementary Material.

## Supplementary material

SUPPLEMENTARY FIGURE S1 Validation of SASP subtyping by different external datasets. **(A)** Heatmap of SASP subtype-specific signature genes in the external dataset GSE103091. **(B)** Kaplan‒Meier survival curves of the TNBCSASP subtypes in the external dataset GSE103091.

SUPPLEMENTARY FIGURE S2 Patients’ inclusion and exclusion flow chart.
