# Supplementary material for: A senescence-associated signature refines the classification of different modification patterns and characterization of tumor immune microenvironment infiltration in triple-negative breast cancer
Source: Front Pharmacol. 2023 May 11;14:1191910. doi: 10.3389/fphar.2023.1191910 (PMC10213971; doi:10.3389/fphar.2023.1191910)
Supplement: Supplementary file 1 [file Table1.DOCX]

**Table S1. The lists of 125 senescence-associated gene set.**

| **Gene(human)** | **Classification** | **State** |
| --- | --- | --- |
| ACVR1B | Transmembrane signal receptors | Transmembrane |
| ANG | Miscellaneous | Secreted |
| ANGPT1 | Intercellular signal molecule | Secreted |
| ANGPTL4 | Intercellular signal molecule | Secreted |
| AREG | Growth factor | Intracellular |
| AXL | Transmembrane signal receptors | Transmembrane |
| NGFRAP1 | Miscellaneous | Intracellular |
| BMP2 | Growth factor | Secreted |
| BMP6 | Growth factor | Secreted |
| C3 | Protease inhibitors | Secreted |
| CCL1 | Cytokine/Chemokine | Secreted |
| CCL13 | Cytokine/Chemokine | Secreted |
| CCL16 | Cytokine/Chemokine | Secreted |
| CCL2 | Cytokine/Chemokine | Secreted |
| CCL20 | Cytokine/Chemokine | Secreted |
| CCL24 | Cytokine/Chemokine | Secreted |
| CCL26 | Cytokine/Chemokine | Secreted |
| CCL3 | Cytokine/Chemokine | Secreted |
| CCL3L3 | Cytokine/Chemokine | Secreted |
| CCL4 | Cytokine/Chemokine | Secreted |
| CCL5 | Cytokine/Chemokine | Secreted |
| CCL7 | Cytokine/Chemokine | Secreted |
| CCL8 | Cytokine/Chemokine | Secreted |
| CD55 | Miscellaneous | Secreted |
| CD9 | Transmembrane signal receptors | Transmembrane |
| CSF1 | Cytokine/Chemokine | Secreted |
| CSF2 | Cytokine/Chemokine | Secreted |
| CSF2RB | Transmembrane signal receptors | Transmembrane |
| CST4 | Protease inhibitors | Secreted |
| CTNNB1 | Transcription factors and regulators | Transmembrane |
| CTSB | (Metallo-)proteases | Secreted |
| CXCL1 | Cytokine/Chemokine | Secreted |
| CXCL10 | Cytokine/Chemokine | Secreted |
| CXCL12 | Cytokine/Chemokine | Secreted |
| CXCL16 | Cytokine/Chemokine | Secreted |
| CXCL2 | Cytokine/Chemokine | Secreted |
| CXCL3 | Cytokine/Chemokine | Secreted |
| CXCL8 | Cytokine/Chemokine | Secreted |
| CXCR2 | Cytokine/Chemokine | Transmembrane |
| DKK1 | Intercellular signal molecule | Secreted |
| EDN1 | Intercellular signal molecule | Secreted |
| EGF | Transmembrane signal receptors | Transmembrane |
| EGFR | Transmembrane signal receptors | Transmembrane |
| EREG | Growth factor | Secreted |
| ESM1 | Intercellular signal molecule | Secreted |
| ETS2 | Transcription factors and regulators | Intracellular |
| FAS | Transmembrane signal receptors | Transmembrane |
| FGF1 | Growth factor | Secreted |
| FGF2 | Growth factor | Secreted |
| FGF7 | Growth factor | Secreted |
| GDF15 | Growth factor | Secreted |
| GEM | Miscellaneous | Intracellular |
| GMFG | Intercellular signal molecule | Intracellular |
| HGF | (Metallo-)proteases | Secreted |
| HMGB1 | Transcription factors and regulators | Intracellular |
| ICAM1 | Miscellaneous | Intracellular |
| ICAM3 | Miscellaneous | Intracellular |
| IGF1 | Growth factor | Secreted |
| IGFBP1 | Protease inhibitors | Secreted |
| IGFBP2 | Protease inhibitors | Secreted |
| IGFBP3 | Protease inhibitors | Secreted |
| IGFBP4 | Protease inhibitors | Secreted |
| IGFBP5 | Protease inhibitors | Secreted |
| IGFBP6 | Protease inhibitors | Secreted |
| IGFBP7 | Miscellaneous | Secreted |
| IL10 | Cytokine/Chemokine | Secreted |
| IL13 | Cytokine/Chemokine | Secreted |
| IL15 | Cytokine/Chemokine | Secreted |
| IL18 | Cytokine/Chemokine | Secreted |
| IL1A | Cytokine/Chemokine | Secreted |
| IL1B | Cytokine/Chemokine | Secreted |
| IL2 | Cytokine/Chemokine | Secreted |
| IL32 | Cytokine/Chemokine | Secreted |
| IL6 | Cytokine/Chemokine | Secreted |
| IL6ST | Transmembrane signal receptors | Transmembrane |
| IL7 | Cytokine/Chemokine | Secreted |
| INHA | Growth factor | Secreted |
| IQGAP2 | Miscellaneous | Intracellular |
| ITGA2 | Transmembrane signal receptors | Transmembrane |
| ITPKA | Protein modifying enzymes | Intracellular |
| JUN | Transcription factors and regulators | Intracellular |
| KITLG | Growth factor | Intracellular |
| LCP1 | Miscellaneous | Intracellular |
| MIF | Protein modifying enzymes | Secreted |
| MMP1 | (Metallo-)proteases | Secreted |
| MMP10 | (Metallo-)proteases | Secreted |
| MMP12 | (Metallo-)proteases | Secreted |
| MMP13 | (Metallo-)proteases | Secreted |
| MMP14 | (Metallo-)proteases | Intracellular |
| MMP2 | (Metallo-)proteases | Secreted |
| MMP3 | (Metallo-)proteases | Secreted |
| MMP9 | (Metallo-)proteases | Secreted |
| NAP1L4 | Miscellaneous | Intracellular |
| NRG1 | Growth factor | Secreted |
| PAPPA | (Metallo-)proteases | Secreted |
| PECAM1 | Miscellaneous | Intracellular |
| PGF | Growth factor | Secreted |
| PIGF | Protein modifying enzymes | Transmembrane |
| PLAT | (Metallo-)proteases | Secreted |
| PLAU | (Metallo-)proteases | Secreted |
| PLAUR | Transmembrane signal receptors | Transmembrane |
| PTBP1 | Miscellaneous | Intracellular |
| PTGER2 | Transmembrane signal receptors | Transmembrane |
| PTGES | Protein modifying enzymes | Intracellular |
| RPS6KA5 | Protein modifying enzymes | Intracellular |
| SCAMP4 | Miscellaneous | Intracellular |
| SELPLG | Transmembrane signal receptors | Transmembrane |
| SEMA3F | Intercellular signal molecule | Secreted |
| SERPINB4 | Protease inhibitors | Intracellular |
| SERPINE1 | Protease inhibitors | Secreted |
| SERPINE2 | Protease inhibitors | Secreted |
| SPP1 | Cytokine/Chemokine | Secreted |
| SPX | Intercellular signal molecule | Secreted |
| TIMP2 | Protease inhibitors | Secreted |
| TNF | Cytokine/Chemokine | Secreted |
| TNFRSF10C | Transmembrane signal receptors | Transmembrane |
| TNFRSF11B | Transmembrane signal receptors | Transmembrane |
| TNFRSF1A | Transmembrane signal receptors | Transmembrane |
| TNFRSF1B | Transmembrane signal receptors | Transmembrane |
| TUBGCP2 | Miscellaneous | Intracellular |
| VEGFA | Growth factor | Secreted |
| VEGFC | Growth factor | Secreted |
| VGF | Intercellular signal molecule | Secreted |
| WNT16 | Intercellular signal molecule | Secreted |
| WNT2 | Intercellular signal molecule | Transmembrane |

**Table S2. The Clinicopathological characteristics of TNBC patients.**

| **Characteristics** | **Total** | **FAM3B expression** | | ***P* value** |
| --- | --- | --- | --- | --- |
|  | ***N* = 122 (%)** | **High**  ***N* = 82 (%)** | **Low**  ***N* = 40 (%)** |  |
| Age (years) |  |  |  | 0.366 |
| ≤50 | 59(48.4) | 42(51.2) | 17(42.5) |  |
| ＞50 | 63(51.6) | 40(48.8) | 23(57.5) |  |
| Menopausal status |  |  |  |  |
| Premenopausal | 63(51.6) | 44(53.7) | 19(47.5) |  |
| Postmenopausal | 59(48.4) | 38(46.3) | 21(52.5) |  |
| Histologic type |  |  |  | 0.966 |
| IDC | 110(90.2) | 74(90.2) | 36(90.0) |  |
| Others | 12(9.8) | 8(9.8) | 4(10.0) |  |
| Tumor grade |  |  |  | 0.424 |
| I-II | 28(23.0) | 16(19.5) | 12(30.0) |  |
| III | 90(73.8) | 63(76.8) | 27(67.5) |  |
| NA | 4(3.3) | 3(3.7) | 1(2.5) |  |
| cN |  |  |  | 0.815 |
| N0 | 47(38.5) | 31(37.8) | 16(40.0) |  |
| N+ | 75(61.5) | 51(62.2) | 24(60.0) |  |
| HER2 |  |  |  | 0.617 |
| Negative | 51(41.8) | 33(40.2) | 18(45.0) |  |
| Low | 71(58.2) | 49(59.8) | 22(55.0) |  |
| Ki67 (%) |  |  |  | 0.162 |
| ≤30% | 22(18.0) | 12(14.6) | 10(25.0) |  |
| ＞30% | 100(82.0) | 70(85.4) | 30(75.0) |  |

Abbreviations: BCS, Breast conserving surgery; HER2, Human epidermal growth factor receptor 2; IDC, Invasive ductal carcinoma; NA, Not available. **p* < 0.05 was considered statistically significant.

**Table S3. Univariate analysis of possible factors influencing the prognosis of TNBC patients.**

| Clinicopathological characteristics | OS | DFS |
| --- | --- | --- |
|  | ***P* value** | ***P* value** |
| Age ($\boldsymbol{\leq}$50 years vs $\mathbf{>}$50 years) | 0.643 | 0.218 |
| Menopausal status (Premenopausal vs postmenopausal) | 0.239 | 0.422 |
| Histologic type (IDC vs Others) | 0.747 | 0.947 |
| Tumor grade (I-II vs III) | 0.527 | 0.885 |
| cN (N0 vs N+) | 0.071 | **0.021** |
| HER2 (HER2 negative vs HER2 low) | 0.388 | 0.746 |
| Ki-67 (≤30% vs $\mathbf{＞}$30%) | 0.468 | 0.822 |
| FAM3B expression (Low vs High) | **<0.001** | **<0.001** |

Abbreviations: BCS, Breast conserving surgery; DFS, Disease-free survival; HER2, Human epidermal growth factor receptor 2; IDC, Invasive ductal carcinoma; OS, Overall survival; **p* < 0.05 was considered statistically significant.

**Table S4. Multivariate analysis of possible factors influencing the prognosis of TNBC patients.**

| **Clinicopathological characteristics** | **OS** | | |  | **DFS** | | |
| --- | --- | --- | --- | --- | --- | --- | --- |
|  | **HR** | **95% CI** | **P value** |  | **HR** | **95% CI** | **P value** |
| cN |  |  | 0.143 |  |  |  | 0.119 |
| N0 | 1.00 |  |  |  | 1.00 |  |  |
| N+ | 2.55 | 0.73-8.93 |  |  | 2.37 | 1.08-5.24 |  |
| FAM3B expression |  |  | **0.039** |  |  |  | **0.013** |
| Low | 1.00 |  |  |  | 1.00 |  |  |
| High | 4.79 | 1.08-21.21 |  |  | 2.91 | 1.26-6.73 |  |

Abbreviations: CI, Confidence interval; DFS, Disease-free survival; HR, Hazard ratio; OS, Overall survival. **p* < 0.05 was considered statistically significant.
