# Supplementary figures and images for: A senescence-associated signature refines the classification of different modification patterns and characterization of tumor immune microenvironment infiltration in triple-negative breast cancer
Source: Front Pharmacol. 2023 May 11;14:1191910. doi: 10.3389/fphar.2023.1191910 (PMC10213971; doi:10.3389/fphar.2023.1191910)

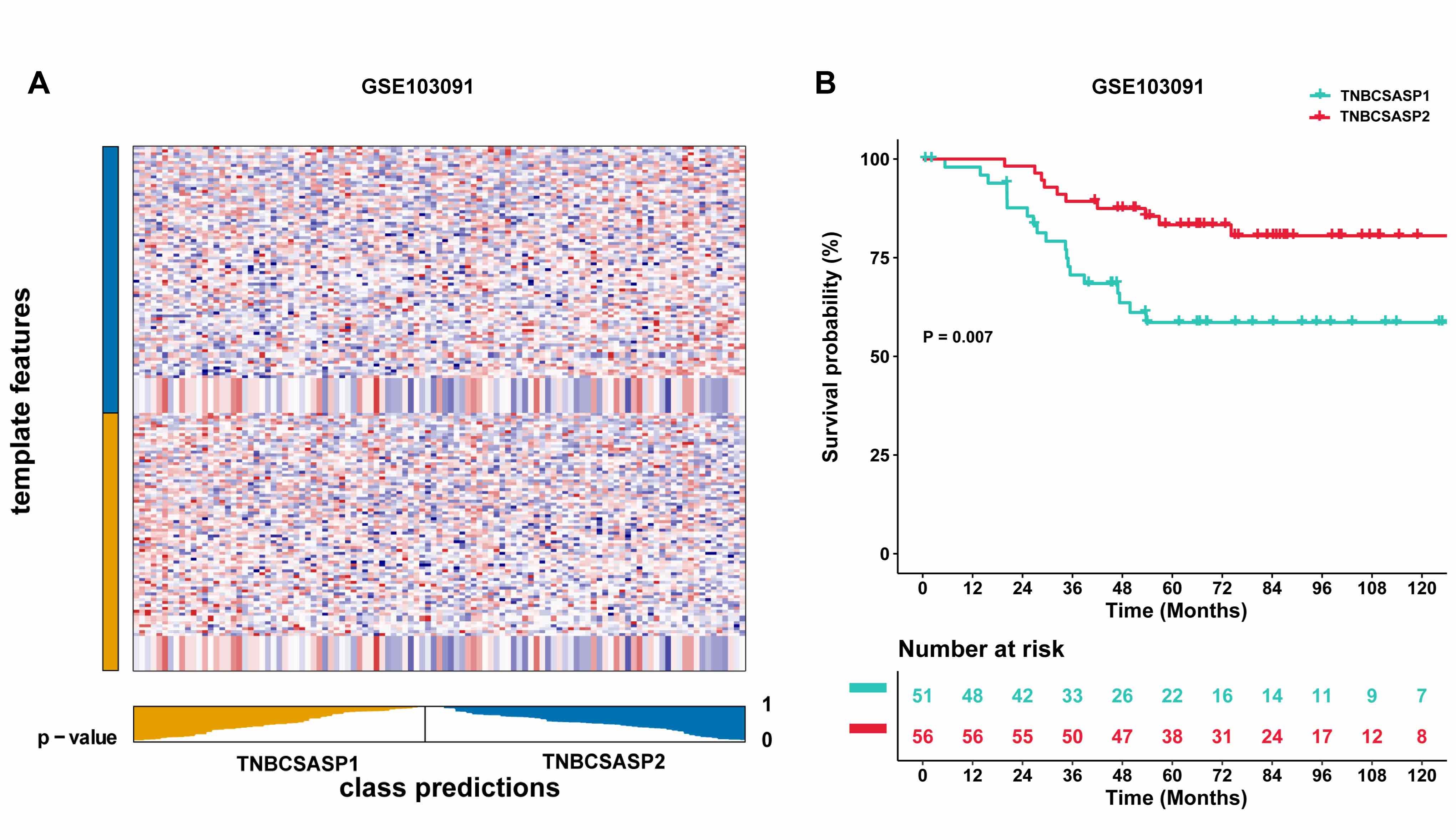

Supplement: Supplementary file 2 [file Image1.jpeg]

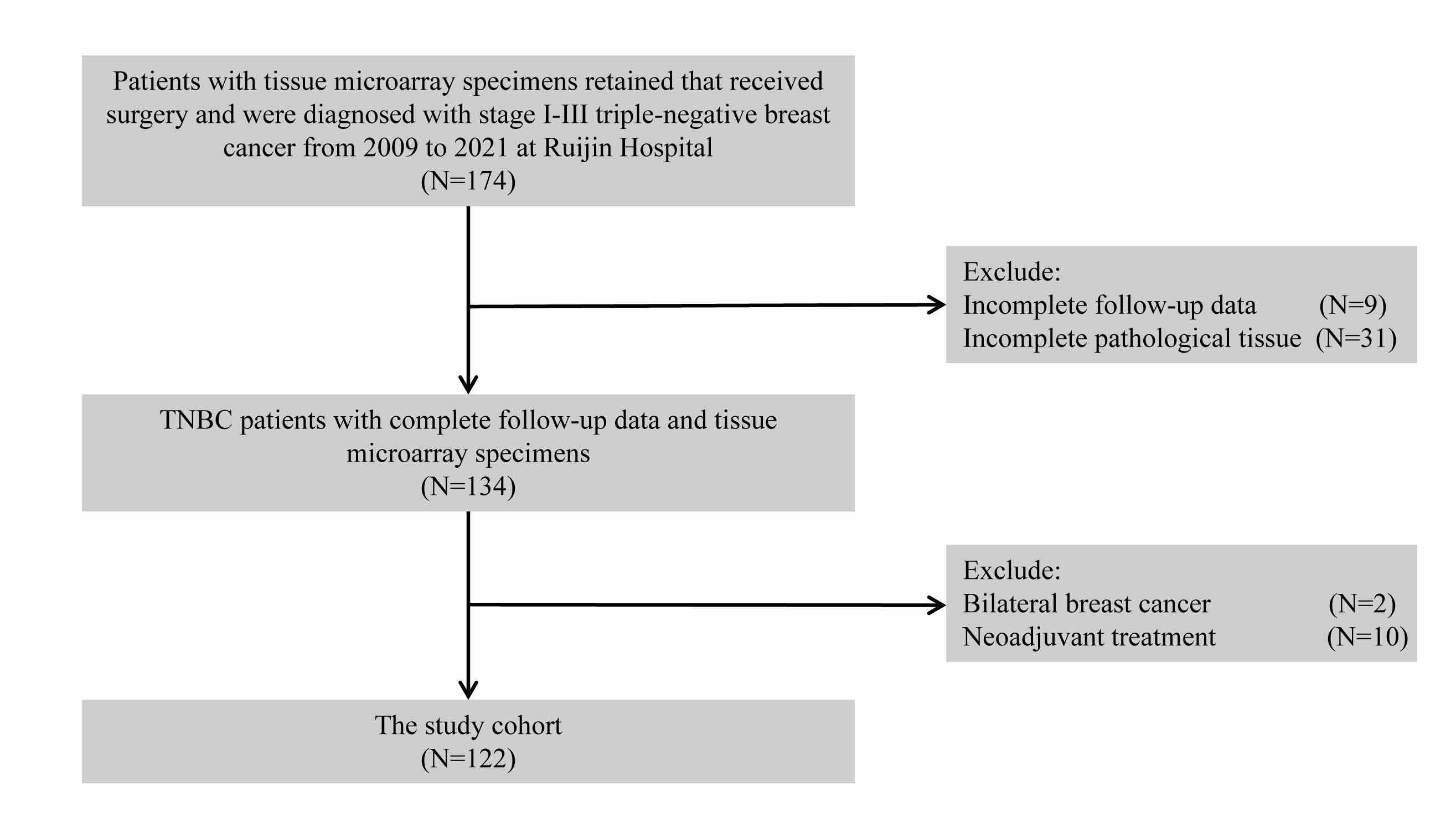

Supplement: Supplementary file 3 [file Image2.jpeg]
