# Supplementary material for: A senescence-associated signature refines the classification of different modification patterns and characterization of tumor immune microenvironment infiltration in triple-negative breast cancer
Source: Front Pharmacol. 2023 May 11;14:1191910. doi: 10.3389/fphar.2023.1191910 (PMC10213971; doi:10.3389/fphar.2023.1191910)
Supplement: Supplementary file 4 [file Table2.DOCX]

**Table S5. Detailed clinicopathological characteristics of TNBC patients.**

| **Patient Number** | **Event of OS** | **Reason of OS** | **Time of OS**  **(month)** | **Event of DFS** | **Reason of DFS** | **Time of DFS**  **(month)** | **Age** | **Menopausalstatus** | **cT** | **cN** | **Pathology** | **Grade** | **HER-2** | **Ki-67** | **Lymphovascular invasion** | **Surgery** | **Chemotherapy** | **Radiotherapy** | **Platinum** |
| --- | --- | --- | --- | --- | --- | --- | --- | --- | --- | --- | --- | --- | --- | --- | --- | --- | --- | --- | --- |
| Patient1 | No | / | 12 | No | / | 12 | ≤50 | Premenopausal | T1 | N+ | IDC | I-II | Low | ≤30% | No | Mastectomy | Yes | Yes | No |
| Patient2 | No | / | 15 | No | / | 15 | ≤50 | Premenopausal | T1 | N+ | IDC | III | Low | >30% | No | Mastectomy | Yes | Yes | No |
| Patient3 | No | / | 18 | No | / | 18 | >50 | Premenopausal | T1 | N0 | IDC | III | Low | >30% | No | Breast conserving surgery | Yes | Yes | No |
| Patient4 | No | / | 12 | No | / | 12 | ≤50 | Premenopausal | T2 | N+ | IDC | III | Negative | >30% | No | Mastectomy | Yes | Yes | No |
| Patient5 | No | / | 13 | No | / | 13 | >50 | Postmenopausal | T1 | N0 | IDC | I-II | Negative | >30% | No | Mastectomy | Yes | No | Yes |
| Patient6 | No | / | 13 | No | / | 13 | >50 | Postmenopausal | T1 | N+ | Others | I-II | Negative | >30% | Yes | Mastectomy | Yes | No | No |
| Patient7 | No | / | 16 | No | / | 16 | ≤50 | Postmenopausal | T2 | N+ | IDC | III | Low | >30% | Yes | Mastectomy | Yes | Yes | Yes |
| Patient8 | No | / | 16 | No | / | 16 | >50 | Postmenopausal | T2 | N0 | IDC | III | Negative | >30% | No | Breast conserving surgery | Yes | Yes | No |
| Patient9 | No | / | 11 | No | / | 11 | >50 | Postmenopausal | T2 | N+ | IDC | III | Low | >30% | No | Mastectomy | Yes | No | Yes |
| Patient10 | No | / | 10 | No | / | 10 | >50 | Postmenopausal | T2 | N+ | IDC | III | Negative | >30% | No | Breast conserving surgery | Yes | Yes | Yes |
| Patient11 | No | / | 19 | No | / | 19 | ≤50 | Premenopausal | T2 | N0 | IDC | III | Negative | >30% | No | Breast conserving surgery | Yes | Yes | No |
| Patient12 | No | / | 19 | No | / | 19 | ≤50 | Premenopausal | T2 | N+ | IDC | III | Negative | >30% | No | Breast conserving surgery | Yes | Yes | No |
| Patient13 | No | / | 10 | No | / | 10 | ≤50 | Premenopausal | T1 | N+ | IDC | III | Negative | >30% | No | Breast conserving surgery | Yes | Yes | Yes |
| Patient14 | No | / | 13 | No | / | 13 | ≤50 | Premenopausal | T2 | N0 | IDC | III | Low | >30% | No | Breast conserving surgery | Yes | Yes | No |
| Patient15 | No | / | 12 | Yes | chest wall recurrence, ipsilateral internal mammary lymph nodes & bone metastases | 6 | ≤50 | Premenopausal | T2 | N+ | IDC | III | Negative | >30% | Yes | Mastectomy | Yes | Yes | No |
| Patient16 | No | / | 15 | No | / | 15 | ≤50 | Premenopausal | T1 | N+ | IDC | III | Low | >30% | Yes | Breast conserving surgery | Yes | Yes | Yes |
| Patient17 | No | / | 15 | No | / | 15 | >50 | Postmenopausal | T1 | N+ | Others | III | Low | >30% | No | Mastectomy | Yes | Yes | Yes |
| Patient18 | No | / | 15 | No | / | 15 | ≤50 | Premenopausal | T2 | N0 | IDC | III | Negative | >30% | No | Mastectomy | Yes | No | No |
| Patient19 | No | / | 11 | No | / | 11 | ≤50 | Premenopausal | T2 | N+ | IDC | III | Low | >30% | No | Mastectomy | Yes | No | Yes |
| Patient20 | No | / | 15 | No | / | 15 | >50 | Postmenopausal | T2 | N0 | IDC | III | Low | >30% | No | Breast conserving surgery | Yes | Yes | Yes |
| Patient21 | No | / | 19 | No | / | 19 | ≤50 | Premenopausal | T2 | N+ | Others | III | Low | >30% | No | Mastectomy | Yes | Yes | No |
| Patient22 | Yes | breast cancer | 4 | Yes | chest wall recurrence & brain metastasis | 2 | ≤50 | Premenopausal | T2 | N+ | Others | III | Low | >30% | No | Mastectomy | Yes | Yes | Yes |
| Patient23 | No | / | 21 | No | / | 21 | >50 | Postmenopausal | T1 | N0 | IDC | III | Negative | >30% | No | Mastectomy | Yes | No | Yes |
| Patient24 | No | / | 17 | No | / | 17 | ≤50 | Premenopausal | T2 | N+ | IDC | III | Low | >30% | No | Breast conserving surgery | Yes | Yes | No |
| Patient25 | No | / | 26 | Yes | recurrence of ipsilateral axillary and supraclavicular lymph nodes & brain metastasis | 18 | >50 | Postmenopausal | T2 | N+ | IDC | III | Low | >30% | No | Mastectomy | Yes | No | No |
| Patient26 | No | / | 26 | Yes | recurrence of ipsilateral axillary and supraclavicular lymph nodes & brain metastasis | 18 | >50 | Postmenopausal | T2 | N+ | IDC | III | Low | >30% | No | Mastectomy | Yes | No | No |
| Patient27 | No | / | 21 | No | / | 21 | >50 | Postmenopausal | T1 | N0 | IDC | III | Low | ≤30% | No | Mastectomy | Yes | No | Yes |
| Patient28 | No | / | 28 | No | / | 28 | >50 | Postmenopausal | T1 | N+ | IDC | III | Negative | >30% | No | Breast conserving surgery | Yes | Yes | Yes |
| Patient29 | No | / | 24 | No | / | 24 | ≤50 | Premenopausal | T2 | N+ | IDC | III | Low | >30% | Yes | Mastectomy | Yes | Yes | Yes |
| Patient30 | No | / | 31 | Yes | liver metastasis | 12 | >50 | Premenopausal | T2 | N+ | IDC | III | Negative | >30% | Yes | Mastectomy | Yes | Yes | Yes |
| Patient31 | No | / | 21 | No | / | 21 | ≤50 | Premenopausal | T3 | N+ | IDC | III | Negative | ≤30% | No | Mastectomy | Yes | Yes | No |
| Patient32 | Yes | pulmonary infection, acute heart failure | 19 | Yes | ipsilateral axillary and supraclavicular lymph nodes | 8 | ≤50 | Premenopausal | T2 | N+ | IDC | III | Negative | >30% | No | Breast conserving surgery | Yes | Yes | Yes |
| Patient33 | No | / | 18 | No | / | 18 | ≤50 | Premenopausal | T1 | N0 | IDC | III | Low | >30% | No | Breast conserving surgery | Yes | Yes | No |
| Patient34 | No | / | 22 | No | / | 22 | >50 | Postmenopausal | T2 | N+ | IDC | III | Low | ≤30% | Yes | Mastectomy | Yes | Yes | Yes |
| Patient35 | No | / | 25 | No | / | 25 | >50 | Premenopausal | T2 | N+ | IDC | III | Low | >30% | Yes | Mastectomy | Yes | Yes | Yes |
| Patient36 | Yes | breast cancer | 9 | Yes | pulmonary & liver metastases | 4 | ≤50 | Premenopausal | T3 | N+ | IDC | III | Low | >30% | Yes | Mastectomy | Yes | No | Yes |
| Patient37 | No | / | 43 | Yes | colon cancer | 25 | >50 | Postmenopausal | T2 | N+ | Others | Unknown | Negative | >30% | No | Mastectomy | Yes | Yes | Yes |
| Patient38 | No | / | 41 | No | / | 41 | ≤50 | Premenopausal | T2 | N+ | Others | Unknown | Low | >30% | No | Breast conserving surgery | Yes | Yes | Yes |
| Patient39 | Yes | breast cancer | 33 | Yes | pulmonary & brain & mediastinal lymph nodes metastases | 17 | ≤50 | Premenopausal | T2 | N+ | IDC | III | Low | >30% | No | Mastectomy | Yes | Yes | Yes |
| Patient40 | No | / | 47 | No | / | 47 | ≤50 | Premenopausal | T2 | N+ | IDC | I-II | Low | >30% | Yes | Mastectomy | Yes | Yes | Yes |
| Patient41 | No | / | 30 | No | / | 30 | ≤50 | Premenopausal | T2 | N+ | IDC | III | Negative | >30% | No | Breast conserving surgery | Yes | Yes | Yes |
| Patient42 | No | / | 37 | No | / | 37 | ≤50 | Premenopausal | T2 | N0 | IDC | III | Low | >30% | Yes | Breast conserving surgery | Yes | Yes | Yes |
| Patient43 | Yes | breast cancer | 14 | Yes | bone & liver metastases | 10 | >50 | Postmenopausal | T2 | N+ | IDC | III | Negative | ≤30% | No | Mastectomy | Yes | Yes | Yes |
| Patient44 | Yes | breast cancer | 12 | Yes | bone & peritoneal lymph node metastasis | 9 | ≤50 | Premenopausal | T2 | N+ | IDC | I-II | Low | >30% | No | Mastectomy | Yes | Yes | No |
| Patient45 | No | / | 53 | No | / | 53 | >50 | Postmenopausal | T2 | N0 | IDC | III | Low | >30% | No | Mastectomy | Yes | No | Yes |
| Patient46 | No | / | 39 | No | / | 39 | >50 | Postmenopausal | T2 | N+ | IDC | III | Low | >30% | No | Mastectomy | Yes | Yes | Yes |
| Patient47 | No | / | 34 | No | / | 34 | ≤50 | Premenopausal | T1 | N+ | IDC | III | Low | >30% | No | Mastectomy | Yes | Yes | Yes |
| Patient48 | No | / | 49 | No | / | 49 | ≤50 | Premenopausal | T1 | N0 | IDC | III | Low | >30% | No | Breast conserving surgery | Yes | Yes | Yes |
| Patient49 | No | / | 46 | No | / | 46 | ≤50 | Premenopausal | T2 | N+ | IDC | III | Negative | >30% | No | Mastectomy | Yes | Yes | Yes |
| Patient50 | No | / | 46 | No | / | 46 | ≤50 | Premenopausal | T2 | N+ | IDC | I-II | Negative | >30% | No | Breast conserving surgery | Yes | Yes | Yes |
| Patient51 | No | / | 38 | No | / | 38 | >50 | Postmenopausal | T2 | N+ | IDC | III | Negative | >30% | No | Mastectomy | Yes | No | Yes |
| Patient52 | No | / | 41 | Yes | contralateral breast cancer | 29 | >50 | Postmenopausal | T2 | N0 | IDC | III | Low | >30% | No | Mastectomy | Yes | Yes | No |
| Patient53 | No | / | 53 | No | / | 53 | >50 | Postmenopausal | T2 | N+ | IDC | III | Negative | >30% | No | Breast conserving surgery | Yes | Yes | Yes |
| Patient54 | Yes | respiratory failure | 16 | Yes | respiratory failure | 16 | >50 | Postmenopausal | T2 | N+ | IDC | I-II | Negative | ≤30% | No | Mastectomy | Yes | Yes | No |
| Patient55 | No | / | 36 | No | / | 36 | >50 | Postmenopausal | T2 | N0 | IDC | III | Low | >30% | No | Mastectomy | Yes | Yes | Yes |
| Patient56 | No | / | 35 | No | / | 35 | >50 | Postmenopausal | T1 | N0 | IDC | III | Negative | >30% | No | Mastectomy | Yes | No | Yes |
| Patient57 | No | / | 45 | No | / | 45 | >50 | Postmenopausal | T2 | N+ | IDC | III | Low | >30% | No | Mastectomy | Yes | No | Yes |
| Patient58 | No | / | 51 | Yes | contralateral breast cancer | 31 | ≤50 | Premenopausal | T2 | N0 | IDC | III | Negative | >30% | No | Mastectomy | Yes | No | No |
| Patient59 | No | / | 36 | No | / | 36 | >50 | Postmenopausal | T2 | N0 | Others | Unknown | Negative | >30% | No | Mastectomy | Yes | No | Yes |
| Patient60 | Yes | breast cancer | 20 | Yes | chest wall recurrence | 9 | >50 | Postmenopausal | T2 | N+ | IDC | III | Low | >30% | Yes | Mastectomy | Yes | Yes | No |
| Patient61 | No | / | 52 | No | / | 52 | ≤50 | Premenopausal | T2 | N0 | IDC | I-II | Negative | >30% | No | Breast conserving surgery | Yes | Yes | Yes |
| Patient62 | No | / | 49 | No | / | 49 | >50 | Postmenopausal | T1 | N0 | IDC | I-II | Low | ≤30% | No | Mastectomy | Yes | No | Yes |
| Patient63 | No | / | 52 | Yes | brain metastasis | 28 | >50 | Postmenopausal | T2 | N+ | IDC | III | Negative | >30% | No | Breast conserving surgery | Yes | Yes | No |
| Patient64 | Yes | breast cancer | 19 | Yes | metastases of bone, lung and liver & non-ipsilateral chest wall,regional lymph nodes | 18 | >50 | Postmenopausal | T2 | N+ | IDC | I-II | Low | >30% | No | Mastectomy | Yes | Yes | No |
| Patient65 | No | / | 60 | No | / | 60 | >50 | Postmenopausal | T1 | N0 | IDC | I-II | Low | ≤30% | No | Mastectomy | Yes | No | Yes |
| Patient66 | No | / | 51 | No | / | 51 | >50 | Postmenopausal | T2 | N0 | IDC | III | Low | >30% | No | Mastectomy | Yes | No | Yes |
| Patient67 | No | / | 59 | No | / | 59 | ≤50 | Premenopausal | T1 | N+ | IDC | III | Low | >30% | No | Mastectomy | Yes | Yes | Yes |
| Patient68 | No | / | 60 | No | / | 60 | ≤50 | Premenopausal | T2 | N+ | IDC | III | Low | >30% | Yes | Mastectomy | Yes | Yes | Yes |
| Patient69 | No | / | 60 | No | / | 60 | >50 | Postmenopausal | T2 | N0 | IDC | III | Negative | >30% | No | Mastectomy | Yes | No | Yes |
| Patient70 | No | / | 51 | No | / | 51 | ≤50 | Premenopausal | T1 | N+ | IDC | III | Negative | >30% | No | Mastectomy | Yes | Yes | Yes |
| Patient71 | Yes | aortic dissection | 5 | Yes | aortic dissection | 5 | >50 | Postmenopausal | T2 | N0 | IDC | III | Low | >30% | No | Breast conserving surgery | Yes | Yes | No |
| Patient72 | Yes | breast cancer | 15 | Yes | distant metastasis | 15 | >50 | Postmenopausal | T3 | N0 | IDC | III | Negative | >30% | No | Mastectomy | Yes | Yes | No |
| Patient73 | Yes | breast cancer | 33 | Yes | chest wall recurrence & pulmonary metastasis | 11 | >50 | Postmenopausal | T3 | N+ | IDC | III | Low | ≤30% | Yes | Mastectomy | Yes | Yes | No |
| Patient74 | No | / | 52 | No | / | 52 | ≤50 | Premenopausal | T1 | N0 | IDC | III | Low | >30% | No | Mastectomy | Yes | No | Yes |
| Patient75 | Yes | breast cancer | 14 | Yes | distant metastasis | 13 | >50 | Postmenopausal | T2 | N0 | IDC | III | Low | >30% | No | Mastectomy | No | No | No |
| Patient76 | Yes | unknown | 16 | Yes | unknown | 11 | ≤50 | Premenopausal | T3 | N+ | IDC | I-II | Low | ≤30% | No | Mastectomy | Yes | Yes | No |
| Patient77 | No | / | 50 | No | / | 50 | >50 | Postmenopausal | T2 | N+ | IDC | III | Low | >30% | No | Mastectomy | Yes | No | Yes |
| Patient78 | No | / | 54 | No | / | 54 | >50 | Postmenopausal | T1 | N+ | IDC | III | Low | >30% | Yes | Mastectomy | Yes | Yes | Yes |
| Patient79 | Yes | breast cancer | 17 | Yes | bone & pulmonary metastases | 15 | >50 | Postmenopausal | T2 | N+ | IDC | I-II | Low | >30% | No | Mastectomy | Yes | Yes | No |
| Patient80 | No | / | 60 | No | / | 60 | ≤50 | Premenopausal | T3 | N+ | IDC | III | Low | ≤30% | No | Mastectomy | Yes | Yes | Yes |
| Patient81 | Yes | breast cancer | 41 | Yes | recurrence of ipsilateral axillary and supraclavicular lymph nodes | 7 | >50 | Postmenopausal | T2 | N+ | Others | I-II | Low | ≤30% | No | Mastectomy | Yes | No | No |
| Patient82 | No | / | 52 | No | / | 52 | ≤50 | Postmenopausal | T2 | N0 | IDC | III | Low | >30% | No | Breast conserving surgery | Yes | Yes | Yes |
| Patient83 | No | / | 52 | No | / | 52 | >50 | Premenopausal | T2 | N0 | IDC | I-II | Low | >30% | No | Mastectomy | Yes | No | Yes |
| Patient84 | No | / | 56 | No | / | 56 | ≤50 | Premenopausal | T2 | N+ | Others | III | Negative | >30% | No | Mastectomy | Yes | Yes | Yes |
| Patient85 | No | / | 60 | Yes | recurrence of non-ipsilateral chest wall,regional lymph nodes | 21 | >50 | Postmenopausal | T2 | N+ | IDC | I-II | Low | >30% | Yes | Mastectomy | Yes | Yes | No |
| Patient86 | No | / | 60 | No | / | 60 | ≤50 | Premenopausal | T2 | N0 | IDC | I-II | Negative | ≤30% | No | Mastectomy | Yes | Yes | Yes |
| Patient87 | No | / | 60 | Yes | recurrence of ipsilateral axillary and supraclavicular lymph nodes | 11 | >50 | Premenopausal | T2 | N+ | IDC | III | Low | >30% | No | Mastectomy | Yes | No | No |
| Patient88 | No | / | 30 | No | / | 30 | ≤50 | Premenopausal | T1 | N0 | Others | Unknown | Negative | >30% | No | Mastectomy | Yes | No | Yes |
| Patient89 | No | / | 60 | No | / | 60 | ≤50 | Postmenopausal | T2 | N+ | IDC | I-II | Negative | ≤30% | No | Mastectomy | Yes | Yes | No |
| Patient90 | No | / | 60 | Yes | bone metastasis | 50 | >50 | Postmenopausal | T2 | N+ | IDC | I-II | Negative | ≤30% | No | Mastectomy | Yes | Yes | Yes |
| Patient91 | No | / | 60 | No | / | 60 | >50 | Postmenopausal | T1 | N0 | IDC | I-II | Negative | >30% | Yes | Mastectomy | Yes | No | No |
| Patient92 | No | / | 60 | Yes | pulmonary & brain metastases | 34 | >50 | Premenopausal | T2 | N+ | IDC | III | Low | >30% | No | Breast conserving surgery | Yes | Yes | No |
| Patient93 | No | / | 12 | No | / | 12 | >50 | Premenopausal | T2 | N+ | IDC | III | Negative | >30% | Yes | Mastectomy | Yes | Yes | Yes |
| Patient94 | No | / | 60 | No | / | 60 | >50 | Postmenopausal | T2 | N0 | IDC | III | Low | >30% | No | Mastectomy | Yes | No | Yes |
| Patient95 | Yes | breast cancer | 35 | Yes | contralateral breast cancer | 22 | ≤50 | Postmenopausal | T2 | N+ | IDC | III | Negative | >30% | Yes | Mastectomy | Yes | No | No |
| Patient96 | No | / | 60 | No | / | 60 | ≤50 | Premenopausal | T3 | N+ | IDC | I-II | Negative | ≤30% | No | Mastectomy | Yes | Yes | No |
| Patient97 | No | / | 60 | No | / | 60 | ≤50 | Premenopausal | T2 | N+ | IDC | III | Low | >30% | No | Mastectomy | Yes | Yes | No |
| Patient98 | No | / | 60 | No | / | 60 | ≤50 | Premenopausal | T3 | N+ | IDC | III | Low | >30% | No | Mastectomy | Yes | Yes | Yes |
| Patient99 | No | / | 60 | No | / | 60 | >50 | Postmenopausal | T1 | N0 | IDC | I-II | Low | ≤30% | No | Mastectomy | Yes | No | No |
| Patient100 | No | / | 14 | No | / | 14 | >50 | Postmenopausal | T2 | N+ | Others | III | Negative | >30% | No | Mastectomy | Yes | Yes | No |
| Patient101 | No | / | 60 | Yes | chest wall recurrence & pulmonary metastasis | 17 | ≤50 | Premenopausal | T2 | N0 | IDC | I-II | Negative | ≤30% | No | Mastectomy | Yes | No | No |
| Patient102 | No | / | 60 | Yes | unknown | 30 | >50 | Postmenopausal | T2 | N0 | IDC | III | Low | ≤30% | No | Mastectomy | Yes | No | No |
| Patient103 | No | / | 60 | No | / | 60 | ≤50 | Premenopausal | T1 | N+ | IDC | I-II | Low | ≤30% | No | Mastectomy | Yes | Yes | No |
| Patient104 | No | / | 6 | No | / | 6 | ≤50 | Premenopausal | T2 | N0 | IDC | III | Negative | >30% | No | Breast conserving surgery | Yes | Yes | No |
| Patient105 | No | / | 8 | No | / | 8 | ≤50 | Premenopausal | T1 | N+ | IDC | III | Low | >30% | No | Mastectomy | Yes | Yes | No |
| Patient106 | No | / | 60 | No | / | 60 | ≤50 | Premenopausal | T2 | N+ | IDC | III | Low | >30% | No | Mastectomy | Yes | Yes | No |
| Patient107 | No | / | 60 | No | / | 60 | >50 | Postmenopausal | T1 | N0 | IDC | I-II | Negative | ≤30% | No | Mastectomy | Yes | Yes | No |
| Patient108 | No | / | 60 | No | / | 60 | >50 | Premenopausal | T1 | N0 | IDC | III | Low | >30% | No | Mastectomy | Yes | No | No |
| Patient109 | No | / | 7 | No | / | 7 | ≤50 | Premenopausal | T1 | N0 | IDC | III | Negative | >30% | No | Breast conserving surgery | Yes | Yes | Yes |
| Patient110 | No | / | 12 | No | / | 12 | ≤50 | Premenopausal | T1 | N0 | IDC | III | Low | >30% | No | Mastectomy | Yes | No | No |
| Patient111 | No | / | 4 | No | / | 4 | >50 | Premenopausal | T2 | N+ | IDC | III | Low | >30% | No | Breast conserving surgery | Yes | Yes | No |
| Patient112 | No | / | 23 | No | / | 23 | >50 | Postmenopausal | T2 | N+ | Others | I-II | Low | ≤30% | Yes | Mastectomy | Yes | Yes | No |
| Patient113 | No | / | 29 | No | / | 29 | >50 | Postmenopausal | T2 | N+ | IDC | I-II | Low | >30% | No | Mastectomy | Yes | No | Yes |
| Patient114 | No | / | 5 | No | / | 5 | ≤50 | Premenopausal | T2 | N0 | IDC | III | Low | >30% | No | Mastectomy | Yes | No | No |
| Patient115 | No | / | 16 | Yes | pulmonary metastasis | 11 | ≤50 | Premenopausal | T1 | N+ | IDC | III | Negative | >30% | Yes | Breast conserving surgery | Yes | Yes | No |
| Patient116 | No | / | 3 | No | / | 3 | >50 | Postmenopausal | T1 | N0 | IDC | III | Negative | >30% | No | Breast conserving surgery | Yes | Yes | No |
| Patient117 | No | / | 20 | Yes | recurrence after breast conserving surgery | 3 | ≤50 | Premenopausal | T2 | N+ | IDC | III | Negative | >30% | No | Breast conserving surgery | Yes | Yes | No |
| Patient118 | No | / | 60 | Yes | metastases of bone, lung, liver and brain | 58 | >50 | Postmenopausal | T2 | N0 | IDC | III | Negative | >30% | No | Mastectomy | Yes | No | Yes |
| Patient119 | No | / | 5 | No | / | 5 | >50 | Postmenopausal | T2 | N0 | IDC | III | Negative | >30% | No | Mastectomy | Yes | No | No |
| Patient120 | No | / | 60 | Yes | recurrence of ipsilateral axillary and supraclavicular lymph nodes | 6 | ≤50 | Premenopausal | T2 | N+ | IDC | III | Low | >30% | Yes | Mastectomy | Yes | Yes | Yes |
| Patient121 | No | / | 2 | No | / | 2 | >50 | Postmenopausal | T1 | N0 | IDC | III | Negative | >30% | No | Breast conserving surgery | Yes | Yes | Yes |
| Patient122 | No | / | 17 | No | / | 17 | ≤50 | Postmenopausal | T1 | N0 | IDC | I-II | Low | >30% | No | Breast conserving surgery | Yes | Yes | No |
